# A cell-permeable probe for the labelling of a bacterial glycosyltransferase and virulence factor†

**DOI:** 10.1039/d3cb00092c

**Published:** 2023-10-19

**Authors:** Yong Xu, Gerd K. Wagner

**Affiliations:** a Department of Chemistry, King's College London UK; b School of Pharmacy, Queen's University Belfast, Medical Biology Centre, 97 Lisburn Road Belfast BT9 7BL UK g.wagner@qub.ac.uk

## Abstract

Chemical probes for bacterial glycosyltransferases are of interest for applications such as tracking of expression levels, and strain profiling and identification. Existing probes for glycosyltransferases are typically based on sugar-nucleotides, whose charged nature limits their applicability in intact cells. We report the development of an uncharged covalent probe for the bacterial galactosyltransferase LgtC, and its application for the fluorescent labelling of this enzyme in recombinant form, cell lysates, and intact cells. The probe was designed by equipping a previously reported covalent LgtC inhibitor based on a pyrazol-3-one scaffold with a 7-hydroxycoumarin fluorophore. We show that this pyrazol-3-ones scaffold is surprisingly stable in aqueous media, which may have wider implications for the use of pyrazol-3-ones as chemical probes. We also show that the 7-hydroxycoumarin fluorophore leads to an unexpected improvement in activity, which could be exploited for the development of second generation analogues. These results will provide a basis for the development of LgtC-specific probes for the detection of LgtC-expressing bacterial strains.

## Abstract 2

We report a chemical probe for the fluorescent labelling of the bacterial glycosyltransferase LgtC, a known virulence factor in *Haemophilus* and *Neisseria* species, and its application with recombinant protein, cell lysates, and intact cells.

## Graphical abstract

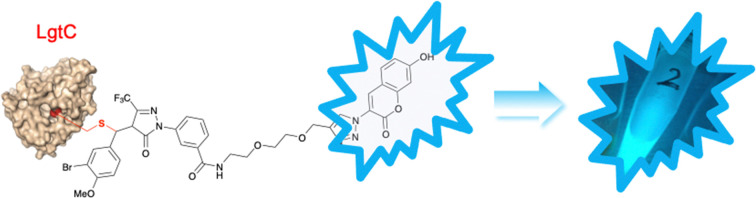

## Introduction

Glycosyltransferases (GTs) are a large family of carbohydrate-active enzymes that are involved in the biosynthesis of complex carbohydrates and glycoconjugates in all domains of life.^1^ GTs catalyse the stereospecific transfer of a mono- or oligosaccharide from a glycosyl donor to an acceptor molecule.^1^ In bacteria, many of the resulting glycosylation products contribute directly to bacterial viability and/or virulence.^2^ Chemical probes for the labelling of bacterial GTs are therefore of great interest to study the role of these enzymes in infection processes and as potential diagnostic tools.

For other carbohydrate-active enzymes such as glycosidases, a considerable number of covalent probes and labelling reagents have been reported, which have found wide application *e.g.*, for proteomics-based profiling and live cell labelling and imaging.^3^ For GTs on the other hand, only a limited number of affinity probes have been reported to date, most of which are substrate analogues based on the UDP-sugar donor.^4^ This lack of chemical probes is particularly striking for bacterial GTs.

LgtC is a retaining α-1,4-galactosyltransferase that catalyses, in certain Gram-negative strains, the formation of a digalactoside motif in the lipooligosaccharide (LOS) portion of the cell envelope.^5^ The resulting digalactoside structures have been associated with bacterial virulence mechanisms in mucosal pathogens, such as increased serum resistance In *Haemophilus influenzae*.^6^ LgtC is therefore an interesting diagnostic and, potentially, therapeutic target for anti-virulence drug discovery.^7^ In contrast to other bacterial GTs, LgtC is structurally and mechanistically well characterised,^5,8^ which may facilitate the development of chemical probes and inhibitors.

**Fig. 1:**
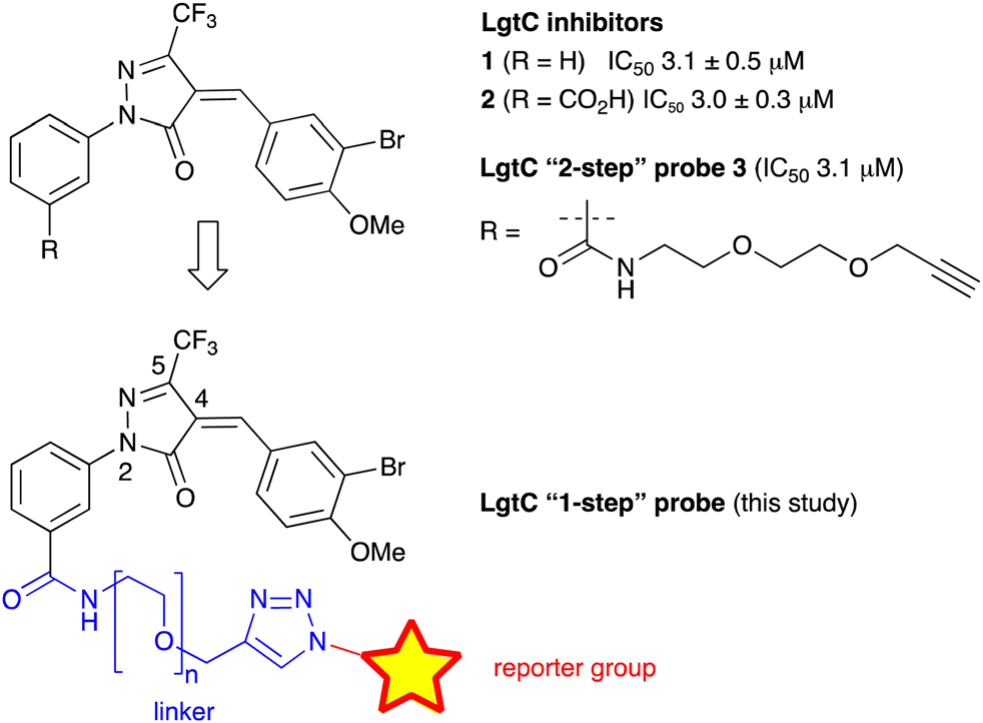
Covalent LgtC inhibitors and probes.

We have recently discovered a novel class of non-substrate-like covalent inhibitors of LgtC based on a pyrazol-3-one scaffold (Fig. 1).^9^ These inhibitors, exemplified by compounds 1 and 2, react with the non-catalytic cysteine residue at position 246 in the LgtC active site *via* a Michael addition at the exocyclic double bond.^9^ We subsequently exploited their covalent mode of action and developed these inhibitors into chemical probes for the fluorescent labelling of LgtC *via* a two-step Click ligation protocol (Fig. 1, compound 3).^3^ Although LgtC has been successfully labelled *via* this two-step approach, the two-step protocol is time consuming, requires relatively large amounts of material, and cannot be readily applied to live cells.

In order to address these limitations and broaden the applicability of our general approach, we have designed a new fluorescent probe for the labelling of LgtC in a single step. Herein, we describe the rational design of the new probe, based on established structure–activity relationships (SAR) around the pyrazol-3-one scaffold, its chemical synthesis, activity towards recombinant LgtC, and successful application for the labelling of recombinant LgtC *in vitro* and in intact cells. We also report results from reactivity and stability tests with prototype inhibitor 1 that address potential concerns around the use of pyrazol-3-ones as chemical tools. Taken together, our findings will provide a basis for the application of this probe to study *e.g.*, expression of LgtC in wild-type strains, mixed cultures, and infection models.

## Results and discussion

### Chemical reactivity and stability of prototype inhibitor 1

Pyrazol-3-ones have previously been reported as PAINS^10^ compounds and may be potentially unstable in aqueous buffers, which raises concerns about their suitability as tool compounds.^11^ To address this concern, we carried out experiments to establish the chemical reactivity and stability of our 5-CF_3_-substituted prototype inhibitor 1.

**Fig. 2:**
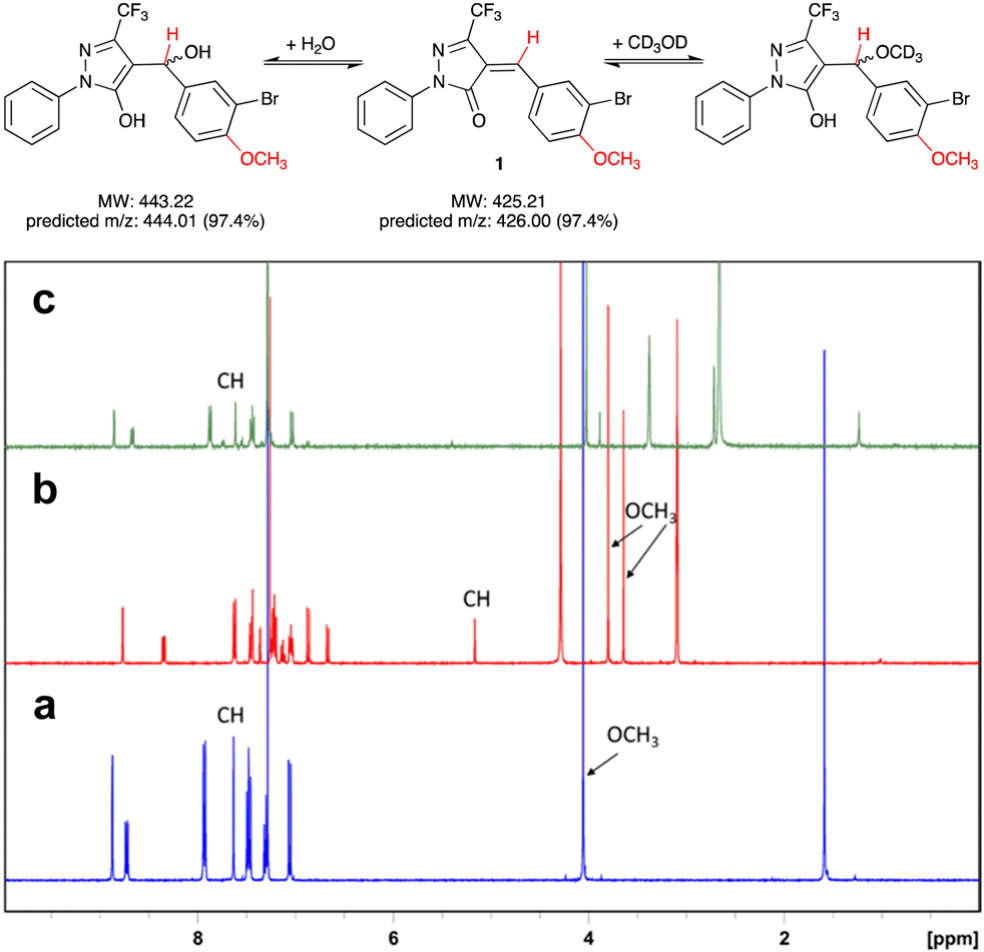
Reversible addition of water or CD_3_OD at the Michael acceptor system of 1. Protons responsible for diagnostic peaks are shown in red. (a) ^1^H-NMR spectrum of 1 in CDCl_3_, (b) after addition of several drops of CD_3_OD, and (c) after dilution (1 : 10) of sample (b) with CDCl_3_. NB that the preferred tautomer upon addition of CD_3_OD to 1 appears to be the enol form (b). All spectra were recorded at 400 MHz.

We first assessed the stability of 1 in protic solvents by thin layer chromatography (TLC) and LC-MS. When a stock solution of 1 in DMSO (50 mM) was diluted in HEPES buffer (pH 7.0) and incubated at room temperature for 10 min, two main spots were observed by TLC. Analysis by LC-MS showed *m*/*z* ratios of 425.9 and 443.9, which correspond closely to the predicted *m*/*z* ratios for, respectively, 1 and its addition product with water (Fig. 2). This suggests that not unexpectedly, 1 can react readily with polar protic solvents, most likely through addition at the Michael acceptor system. Interestingly, when the solvents were evaporated and the crude residue analysed by ^1^H-NMR, the main species was 1, with only a trace amount of the addition product. We therefore hypothesised that the addition reaction of 1 with polar protic solvents is reversible.

To further investigate the reversibility of this putative Michael addition, we used a previously reported NMR-based protocol.^12^1 was dissolved in CDCl_3_ and the ^1^H-NMR spectrum recorded before and after addition of CD_3_OD (Fig. 2a and b). Upon addition of CD_3_OD, the singlet for the methine proton in 1 shifted from 7.65 to 5.15 ppm, and the signal for the 4-methoxy substituent, previously a single singlet at 4.05 ppm, now appeared as two singlets at 3.8 and 3.6 ppm. These changes are diagnostic for the formation of both diastereomers upon addition of CD_3_OD at the double bond in 1 (Fig. 2).

Next, 50 μL of the NMR sample were diluted in 500 μL of CDCl_3_. This resulted in the disappearance of the singlets at 3.6, 3.8, and 5.15 ppm, and the reappearance of the singlets at 4.05 and 7.65 ppm. These results demonstrate that the addition of CD_3_OD at the Michael acceptor system of 1 is indeed reversible.

Equally importantly, no degradation products were observed during these stability experiments. It has been shown previously that under basic conditions, 5-methyl pyrazol-3-ones can undergo a retro-Knoevenagel reaction.^13^ No such reaction was observed for 1 in aqueous buffer. This may be due at least in part to the electron-withdrawing effect of the 5-CF_3_ substituent, which appears to favourably modulate both the stability and reactivity of the pyrazol-3-one scaffold in 1. Importantly for potential protein labelling applications, the reversibility of the Michael addition reaction towards aqueous solvent allows further reaction with alternative nucleophiles such as cysteine. A similar kind of reversible Michael addition has previously also been reported in pyrrolobenzodiazepines (PBDs).^14^ Taken together, these results suggested that 1 is a suitable scaffold for the development of labelling reagents for LgtC.

### Probe design and synthesis

Previous SAR around the pyrazol-3-one scaffold showed that a substituent at the *meta* position of the 2-phenyl ring does not disrupt the covalent interaction with LgtC (Fig. 1).^3,9^ We therefore chose this position for installation of a linker and reporter group, while retaining the 5-CF_3_ and 4-(3-bromo-4-methoxybenzyl) substituents present in our most potent inhibitors (Fig. 1). This flexible design also allowed us to readily vary the length of the linker and the nature of the reporter group.

**Scheme 1:**
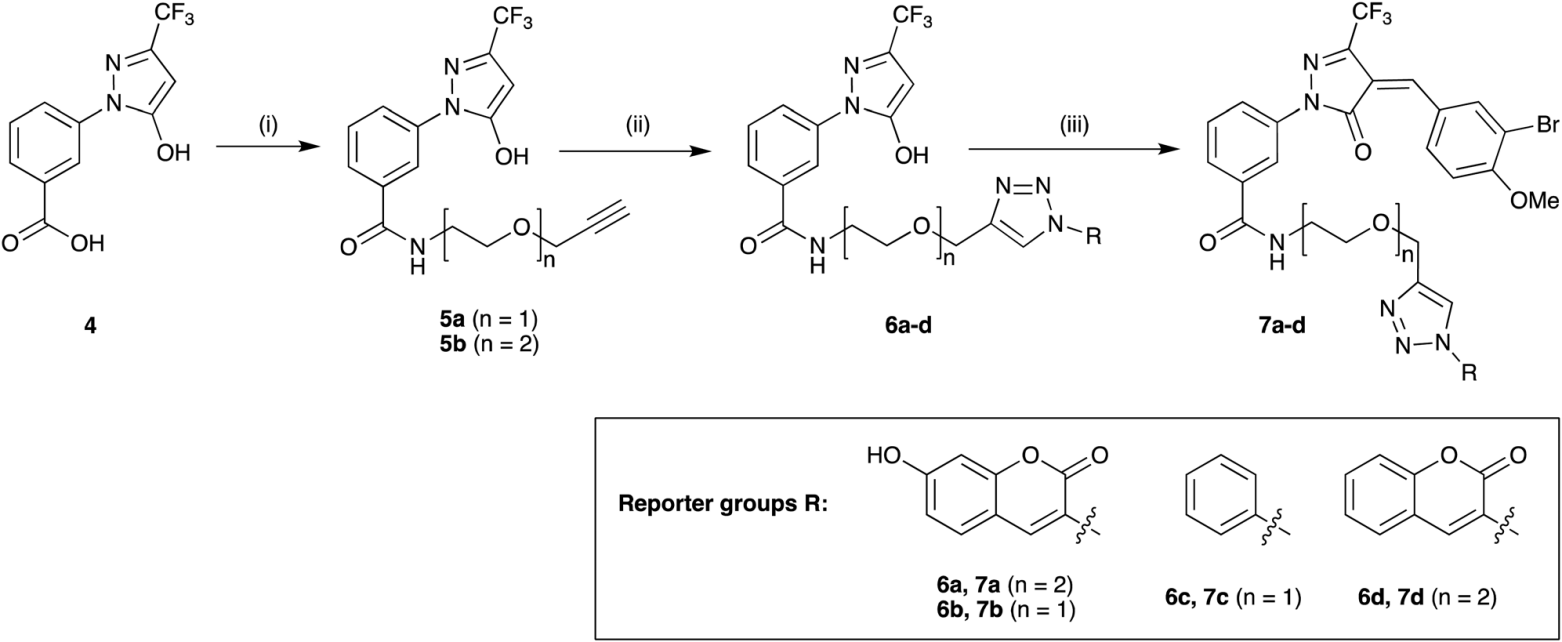
Synthesis of target molecules. Reagents & conditions: (i) 2-(prop-2-yn-1-yloxy)ethan-1-amine (5a) or 2-(2-(prop-2-yn-1-yloxy)ethoxy)ethan-1-amine (5b), HBTU, DIEA, DMF, rt, overnight, 5a (65%) or 5b (64%); (ii) azide, CuSO_4_, sodium ascorbate, *t*-BuOH/water (1 : 1), rt, overnight, 6a (30%), 6b (44%), 6c (63%), or 6d (68%); (iii) 3-bromo-4-methoxybenzaldehyde, 160 °C/microwave, 15 min, 7a (31%), 7b (71%), 7c (60%), or 7d (47%). For synthesis of reporter group azides see ESI.†

Target molecules 7 were prepared in three steps from pyrazol-3-one 4 (Scheme 1). Installation of a short linker *via* amide coupling at the carboxy group gave 5a and 5b as previously reported.^3^ Attempts to react these intermediates in a Knoevenagel condensation with 3-bromo-4-methoxybenzaldehyde remained unsuccessful. Instead, different groups (substituents R, Scheme 1 and Table 1) were introduced directly at 5a and 5b by copper(i)-catalyzed cycloaddition^15^ of the terminal alkyne with the requisite azides under standard conditions. The required azides were readily synthesised using established chemistry (ESI†). In the final step of our synthesis, our primary targets 7a and 7b as well as two analogues 7c and 7d were obtained by microwave-assisted condensation of 6a–d with 3-bromo-4-methoxybenzaldehyde. Several rounds of precipitation and crystallization from hexane/ethyl acetate delivered the final compounds in yields of 31–71%. To study the role of the Michael acceptor system for probe activity, we also synthesised congener 8, which is lacking the exocyclic double bond, as a control compound (Table 1 and ESI,† Scheme S2).

**Table 1:** Inhibition of LgtC^a^

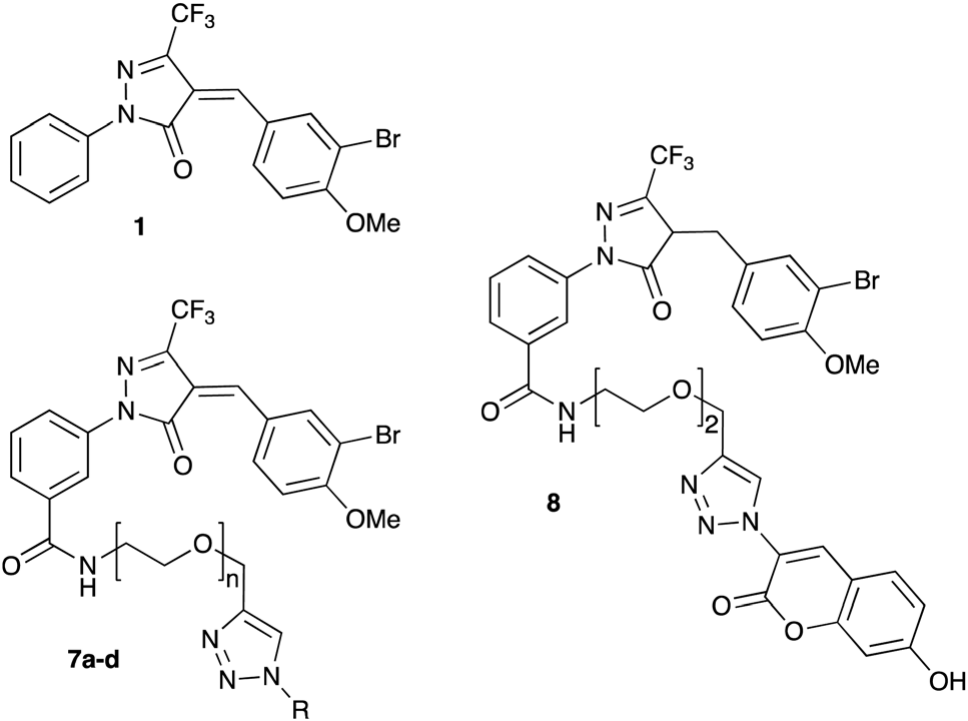				
Cmpd	*n*	*R* (reporter group)	IC_50_ (μM)	Turnover (%)
1	n.a.	n.a	3.1 ± 0.5	32
7a	2	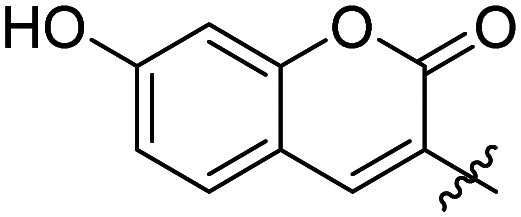	0.099 ± 0.010	32
7b	1	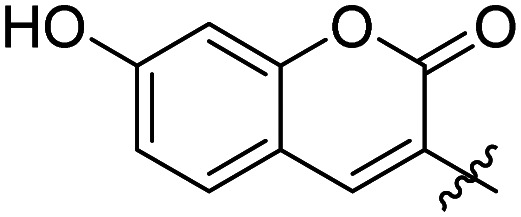	0.107 ± 0.014	34
7c	1	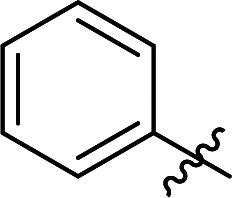	3.6 ± 0.1	23
7d	2	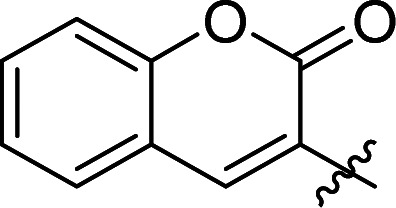	1.5 ± 0.2	37
8	n.a.	n.a.	8.3 ± 0.7	38

a Conditions: LgtC was pre-incubated with test compound, UDP-Gal (28 μM), MnCl_2_ (5 mM), CIP (10 U mL^−1^), CEL (1 mg mL^−1^), Triton (0.01%) for 30 min at 30 °C in 13 mM HEPES buffer (pH 7.0). Lactose (2 mM) was added, and the reactions were incubated for 20 min at 30 °C. Each experiment was carried out in triplicate.

### Fluorescence emission of 7a

As our primary reporter group we had chosen the commonly used fluorophore 7-hydroxycoumarin.^16^ In order to understand if covalent addition of a nucleophile at the Michael acceptor system of our probes or, potentially, at 7-hydroxycoumarin itself may impact fluorescence emission, we recorded fluorescence emission spectra of 7a in aqueous solution, with and without preincubation with cysteine (ESI,† Fig. S1). While maximum fluorescence emission was observed at 477 nm in both cases, interestingly, addition of cysteine resulted in a qualitatively stronger signal. This suggested that disruption of the Michael acceptor system does not adversely affect fluorescence emission.

### *In vitro* inhibition of LgtC

The inhibitory activity of the new pyrazol-3-ones 7 and 8 towards recombinant LgtC was evaluated in a previously established enzyme-coupled assay.^17^ LgtC catalyses the transfer of α-d-galactose from a UDP-α-d-galactose donor to lactose acceptor, with concomitant release of UDP as a secondary product.^5^ The assay uses calf intestinal phosphatase (CIP) to cleave inorganic phosphate from UDP, which is then detected colorimetrically with Malachite Green. LgtC activity is kept at 20–50% to ensure comparability across different assay runs.^17^

Upon preincubation with LgtC for 30 min, the new fluorescent probe 7a showed 30-fold better inhibitory activity in this assay than prototype inhibitor 1 (Table 1 and ESI,† Fig. S2). This result was unexpected, as previous results had suggested that a linker in the meta position does not affect inhibitory activity.^3^ To ensure this surprising result was not an assay artefact, we carried out the requisite control experiments, which confirmed that 7a does neither inhibit CIP, nor interfere with the absorbance readings of the Malachite Green complex (ESI,† Fig. S3).

Next, we tested analogues 7b–d, which differ from 7a in the length of the linker (7b) or the nature of the reporter group (7c, 7d). Interestingly, the length of the linker had no effect on inhibitory activity, whereas replacement of the 7-hydroxycoumarin fluorophore in 7a with a phenyl (7c) or simple coumarin (7d) substituent led to a drop in activity to the levels observed for the parent inhibitor 1 (Table 1). Taken together, these results suggest that the 7-hydroxycoumarin fluorophore, and in particular the 7-hydroxy substituent, contributes to the sub-micromolar activity of the new LgtC probes, whereas the length of linker is of lesser importance. This is consistent with the activity observed for control compound 8 (Table 1 and ESI,† Scheme S2) which is lacking the exocyclic double bond but is otherwise identical to 7a. While 8 is more than 80-fold less active than 7a, it retains similar activity to parent inhibitor 1, despite the absence of the Michael acceptor system. This result suggests that the 7-hydroxycoumarin substituent makes an important non-covalent binding interaction with LgtC, and that both the pyrazol-3-one scaffold and the 7-hydroxycoumarin reporter group are crucial features for the potent activity observed for probes 7a and 7b.

Structurally, these activities can be rationalised with a binding mode that positions the 7-hydroxycoumarin portion in the donor binding site of LgtC, and the Michael acceptor in the proximity of Cys246 (ESI,† Fig. S4), which as previously shown^9*b*^ is the target residue for covalent addition of inhibitors in this series. In this orientation, 7a and 7b can mimic critical interactions between LgtC and a donor analogue in the crystal structure,^5^ including hydrogen bonding with Asp8 and π–π stacking with Tyr11. Analogues lacking the 7-hydroxy group (7d, 7c, 8) can engage in some, but not all of these interactions, which may explain their reduced activity. This interpretation is also consistent with our previous finding that inhibitory activity of LgtC inhibitors such as 1 is driven by non-covalent interaction rather than covalent inactivation.^9*a*^

### Labelling of recombinant LgtC with 7a

**Fig. 3:**
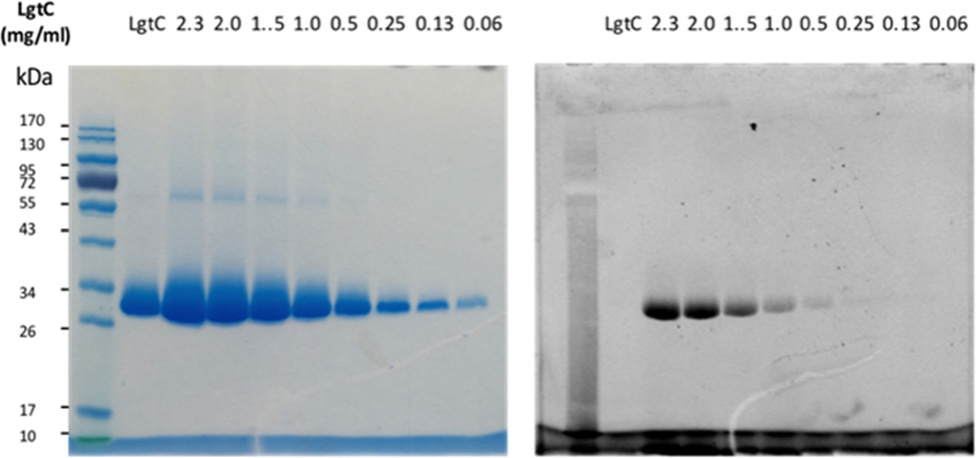
Labelling of recombinant LgtC with 7a. Conditions: 7a (50 μM) was incubated with LgtC for 30 min at 30 °C. Unbound probe was removed by Vivaspin concentrators (cut-off: 10 kDa). The protein sample was quantified by Bradford assay, serially diluted, and denatured at 100 °C for 10 min. Samples were analysed by 12% SDS-PAGE and visualised by Coomassie staining (left) and in-gel fluorescence scanning (right).

Although attachment of the fluorophore was not originally expected to affect the interaction of the probe with LgtC, we reasoned that the enhanced binding affinity for LgtC may actually be an advantage for labelling applications. To examine the labelling of recombinant LgtC by 7a, the enzyme was incubated with the probe at 30 °C for 30 min, unbound probe was removed by centrifugation, and the residue was washed with HEPES buffer (13 mM, pH 7.0). The labelled protein was quantified, serially diluted (0.06–2.3 mg mL^−1^), denatured, and analysed by gel electrophoresis (Fig. 3). Visualisation by in-gel fluorescence and Coomassie staining showed a band at 32 kDa, as expected for LgtC. At 0.5 mg mL^−1^, the minimal concentration of LgtC that could be detected was about 10-fold higher by fluorescence emission than by staining (0.06 mg mL^−1^). We attributed the limited sensitivity to the harsh denaturing conditions, which may have resulted in the cleavage of the covalent bond between protein and probe.

**Fig. 4:**
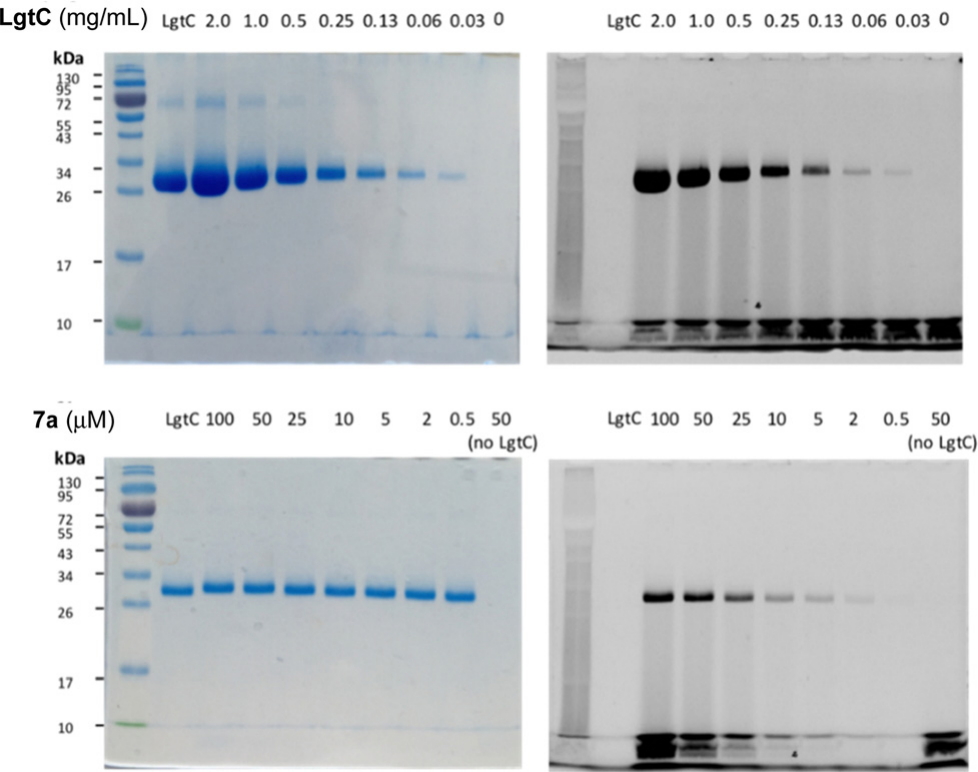
Optimisation of labelling protocol for recombinant LgtC with 7a. Conditions: Top row: 7a (50 μM) was incubated with variable concentrations of LgtC (0–2.0 mg mL^−1^). Bottom row: variable concentrations of 7a (0.5–100 μM) were incubated with LgtC (0.25 mg mL^−1^). Samples were analysed by 12% SDS-PAGE and visualised by Coomassie staining (left) and in-gel fluorescence scanning (right).

To avoid cleavage of LgtC-7a conjugates, we next investigated milder denaturation conditions. Since the reaction mixture of LgtC and 7a was incubated at 30 °C, we chose this temperature as a starting point. Pleasingly, preliminary results under these conditions showed significantly improved sensitivity and allowed us to determine the LgtC detection limit of 7a as well as the optimal probe concentration. When LgtC at different concentrations (0–2.0 mg mL^−1^) was incubated with 7a (50 μM) under these milder conditions, the minimal concentration of LgtC that could be visualised by fluorescence scanning was 0.03 mg mL^−1^ (Fig. 4, top). Next, a fixed amount of LgtC (0.25 mg mL^−1^) was incubated with various concentrations of 7a (0.5–100 μM) for 20 min, showing detectable levels of LgtC down to a concentration of 2 μM 7a (Fig. 4, bottom).

In these experiments, we also further simplified the labelling protocol by avoiding the concentration step and leaving unbound probe in the sample during gel electrophoresis. This adjustment is advantageous as it allows the labelling experiment to be miniaturised to a total volume of 10 μL or even less, while a larger amount of protein sample is necessary for the protocol with centrifugation. Taken together, these experiments demonstrated not only the concentration-dependency of LgtC labelling with 7a, but also established a robust protocol for labelling experiments in more complex samples, including information about relevant detection limits.

### Labelling of LgtC in cell lysates and intact cells with 7a

Having established that probe 7a can label purified recombinant LgtC, we examined its capacity to label cell lysates from a LgtC-expressing *E. coli* clone. This clone has previously been transformed with a plasmid containing sequences encoding His-tagged LgtC and ampicillin resistance.^18^ LgtC expression in cells was induced with isopropyl-β-d-thiogalactopyranoside (control: no induction). After cell disruption, cell lysates were centrifuged, and the supernatant was collected and incubated with 7a at final concentrations of 10, 25, and 50 μM.

**Fig. 5:**
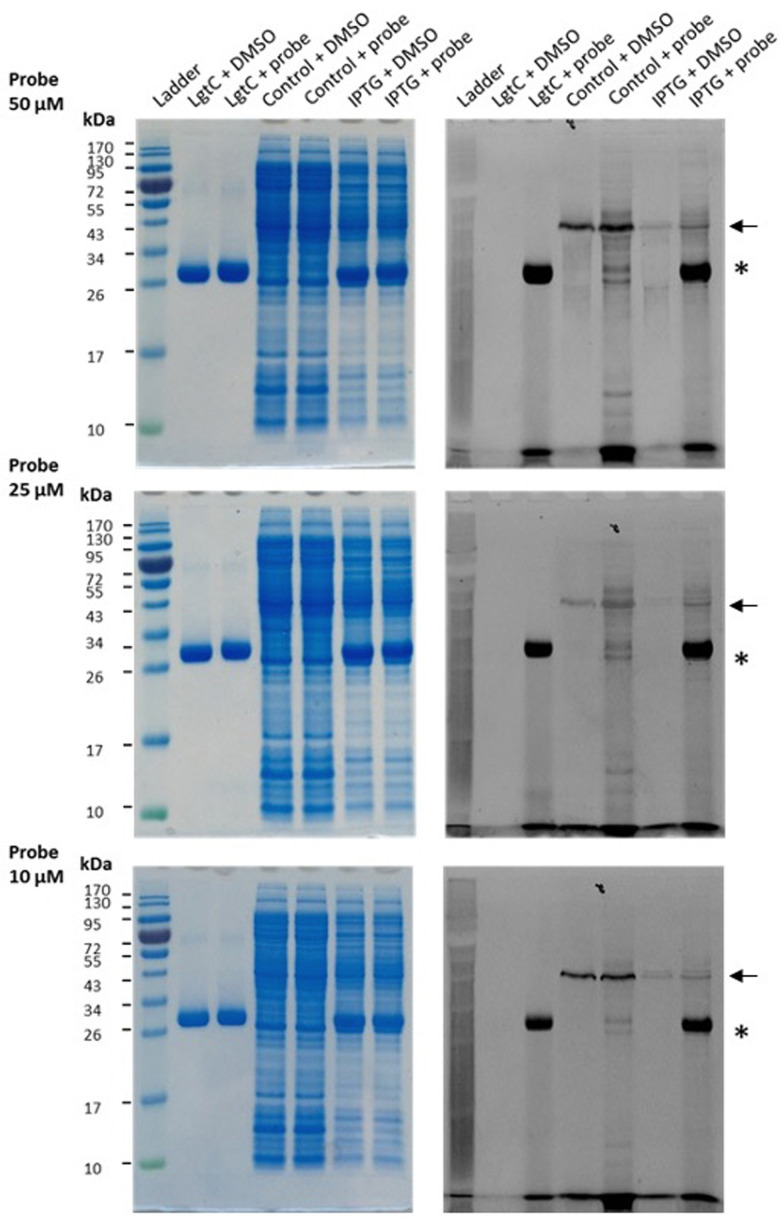
Labelling of LgtC in cell lysates with 7a. Conditions: the soluble proteome from *E. coli* clone NMC-41 following induction with IPTG (lanes “IPTG”) or no induction (lanes “control”) was incubated with 7a at 10, 25 or 50 μM. Samples were analysed by 12% SDS-PAGE and bands were visualised by Coomassie Blue staining (left) and in-gel fluorescence scanning (right). The asterisk indicates the LgtC band; the arrow indicates the putative Ef-Tu band. Recombinant LgtC was included for reference (lanes “LgtC”). Experiments were carried out in duplicate, representative results are shown.

Analysis by SDS-PAGE and visualisation by fluorescence emission showed successful labelling of LgtC at all three concentrations of 7a (Fig. 5). Not unexpectedly, labelling of a small number of additional proteins was also observed (lane 5 & lane 7), which may be due to a certain degree of promiscuous reactivity of the Michael acceptor system. The strongest off-target band was observed at ∼45 kDa. Preliminary results from protein mass spectrometry suggest that this band may correspond to the bacterial elongation factor Ef-Tu (unpublished results). This is not surprising, as Ef-Tu is the most abundant protein in bacterial cells and possesses a non-catalytic cysteine in the substrate binding site, which is capable of reacting with 7a. It is notable, however, that in lysates from non-IPTG-induced cells (lane 5), labelling was observed of a ∼32 kDa protein band, which presumably is due to leaky expression of LgtC. This shows that 7a was still able to label LgtC even though it was not the most abundant protein in this sample, indicating that labelling capacity is driven not only by protein abundance but also by at least a certain degree of specificity.

Finally, we investigated the applicability of 7a for labelling of LgtC in intact cells. Most existing probes for GTs are based on a sugar-nucleotide scaffold, whose negatively charged diphosphate moiety limits penetration of the bacterial membrane.^4^ We speculated that due to its uncharged nature, 7a may in contrast be able to penetrate into bacterial cells.

To identify any potential effect of 7a on bacterial viability, we first monitored growth of a *E. coli* DH5α culture in the presence or absence of probe. No significant effect on bacterial growth was observed at both concentrations tested (25 μM, 50 μM) over a period of 6 h (ESI,† Fig. S5). These results suggest that the probe does not affect bacterial viability and is indeed suitable for labelling of intact cells.

**Fig. 6:**
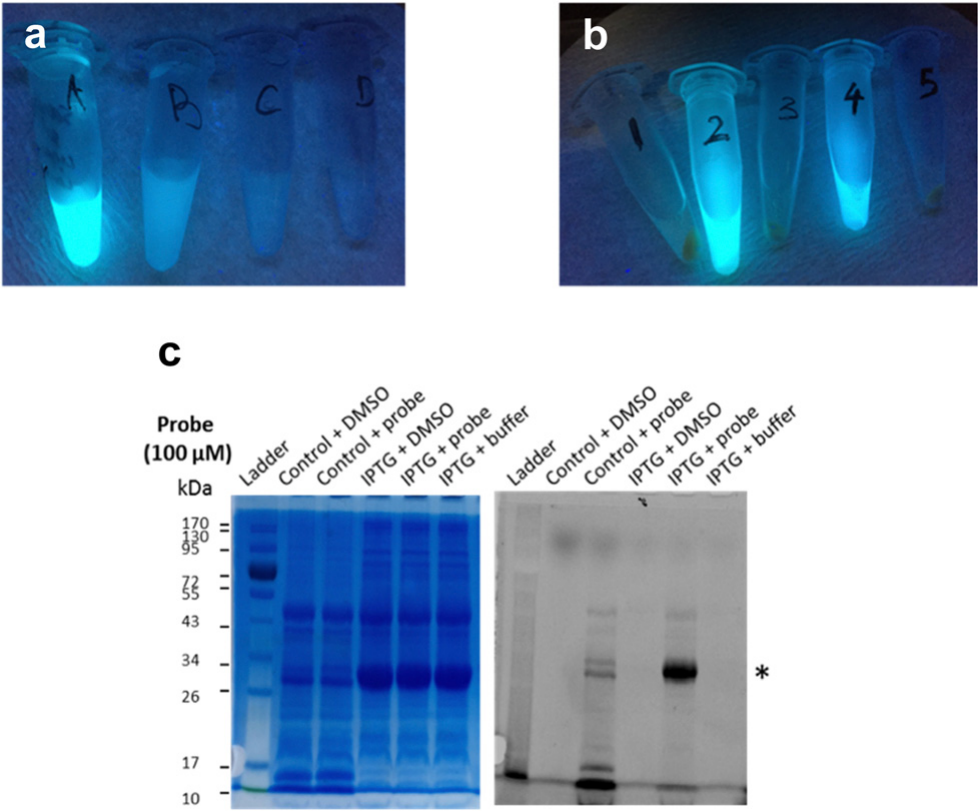
Labelling of LgtC in intact *E. coli* cells with 7a. (a) The supernatant of cells treated with 7a shows progressively weaker fluorescence emission (*λ*_ex_ 366 nm) after 1 h incubation (A), 1st wash with DMSO (10%) in HEPES buffer (B), 2nd wash with HEPES buffer (C), and 3rd wash with HEPES buffer. (b) Strong fluorescence emission (*λ*_ex_ 366 nm) was observed in lysates of intact *E. coli* cells incubated with 7a (100 μM) with (4) and without (2) prior IPTG induction of LgtC expression, but not in control samples treated with DMSO (3: with IPTG, 1: without IPTG) or HEPES buffer (5: with IPTG) instead of 7a. (c) The supernatant of the five samples in (b) was analysed by 12% SDS-PAGE and bands visualised by Coomassie Blue staining (left) and in-gel fluorescence scanning (right). The asterisk indicates the LgtC band.

Next, frozen cell samples that had been induced with IPTG (control: no IPTG induction) were suspended in HEPES buffer and incubated with 7a (control: DMSO only) for 1h at 30 °C, with gentle shaking every 10 min. To remove any unbound probe, cells were washed consecutively with DMSO (10%) in HEPES buffer (1×) and HEPES buffer only (2×) and centrifuged. No residual fluorescence was observed in the supernatant following these wash steps (Fig. 6a). To lyse the cells, the cell pellet was re-suspended in BugBuster Buffer, shaken on a rotating mixer, and centrifuged. Supernatant from samples incubated with 7a showed very strong fluorescence emission (Fig. 6b, samples 2 and 4), whereas control samples did not (Fig. 6b, samples 1, 3 and 5), which suggests that 7a can indeed penetrate the bacterial membrane.

To confirm that 7a can label LgtC in intact cells under these conditions, samples 1–4 were separated by SDS-PAGE and analysed by Coomassie blue staining and in-gel fluorescence scanning (Fig. 6c). Pleasingly, the labelling profiles obtained from this experiment with intact cells was similar to those observed with cell lysates. Fluorescent labelling of the LgtC band is evident in both samples that had been treated with 7a. As expected, the fluorescent band is particularly strong where LgtC expression had been induced with IPTG (Fig. 6c, lane 5). However, as in the cell lysate experiments, it is also visible in the control sample without IPTG induction, which can be attributed to leaky expression of LgtC (Fig. 6c, lane 3). While LgtC was not the only protein labelled under these conditions, these results confirm that 7a can label LgtC even at low levels not only in cell lysates, but also in intact cells.

## Experimental

### Chemistry

Detailed synthetic procedures and compound characterisation data are reported in the supplementary information.

### Reversible Michael addition at compound 1

1 (10 mg, 0.023 mmol) was dissolved in CDCl_3_ in a standard NMR tube. The ^1^H NMR spectrum was acquired before and after addition of several drops of CD_3_OD. An aliquot (50 μL) of the solution was combined with CDCl_3_ (500 μL) in another NMR tube and the ^1^H NMR spectrum was recorded again on a Bruker BioSpin at 400 MHz.

### Protein expression

LgtC was expressed and purified as previously described.^19^ Recombinant LgtC was activated with DTT (10 mM in HEPES buffer) in a 1 : 1 ratio for 30 min at 30 °C prior to each experiment, unless otherwise stated.

### LgtC inhibition assays

Enzyme activity and inhibition experiments were carried out using a previously reported colorimetric assay protocol.^17^ Unless otherwise stated, all assays were carried out in Nunc clear, flat-bottom 96 well microplates on a Polarstar Optima microplate reader (BMG Labtech). For IC_50_ experiments, LgtC activity was adjusted to 20–50% turnover of UDP-Gal donor. We have previously shown that within this turnover range IC_50_ values are obtained reproducibly.^17^ All concentrations for the assay components are final concentrations. Aliquots (15 μL each) of activated LgtC, MnCl_2_ (5 mM), CEL (1 mg mL^−1^), CIP (10 U mL^−1^), Triton (0.01%) and HEPES buffer (13 mM, pH 7.0) were combined with inhibitor at various concentrations in DMSO (15 μL, 10% final DMSO concentration) or DMSO only (15 μL, control) in the requisite microplate wells. UDP-Gal donor (15 μL, 28 μM) was added, and the mixtures were pre-incubated for 30 min at 30 °C. Lactose acceptor (30 μL, 2 mM) or HEPES buffer (30 μL, control) were added, and the reactions were incubated for 20 min at 30 °C. Reactions were stopped by addition of Malachite Green reagent A (30 μL). The microplate was shaken carefully, and Malachite Green Reagent B (30 μL) was added. The colour was allowed to develop over 20 min, and the absorbance in each well was recorded at 620 nm. Absorbance measurements were used to calculate enzyme activity as previously described.^17^

### Labelling of recombinant LgtC

Probe 7a (500 μM in DMSO, 100 μL) was incubated with LgtC (0–2.0 mg mL^−1^, 900 μL) at 30 °C for 30 min. Loading buffer (2.5 μL)^3^ was added to each sample and incubated for another 15 min at 30 °C. Samples were separated on a 12% tris-glycine SDS-polyacrylamide electrophoresis gel. Upon completion, the gel was washed quickly with pure water. Protein bands were visualised by in-gel fluorescence scanning on a GE Amersham Imager 600, and by Coomassie staining.

### Labelling of LgtC in cell lysates

*E. coli* LgtC-overexpression clone NMC-41^18^ was cultured as previous described^3^ and cells were harvested by centrifugation (4000 rpm, 4 °C, 10 min). The cell pellet was resuspended in disruption buffer and lysed by sonication. The cell suspension was centrifuged (15 000 rpm, 4 °C, 1 h) and the supernatant was collected and stored at −80 °C prior to the labelling experiment.

Probe 7a (100, 250, and 500 μM in DMSO, 1 μL) was incubated with cell lysate (9 μL) at 30 °C for 30 min. Loading buffer (2.5 μL) was added to each sample and incubated for another 15 min at 30 °C. Samples were separated on a 12% tris-glycine SDS-polyacrylamide electrophoresis gel. Upon completion, the gel was washed quickly with pure water. Protein bands were visualised by in-gel fluorescence scanning on a GE Amersham Imager 600, and by Coomassie staining. Experiments were carried out in duplicate with reproducible results.

### Labelling of intact cells

*E. coli* LgtC-overexpression clone NMC-41^18^ was cultured as previous described^3^ and cells were harvested by centrifugation (4000 rpm, 4 °C, 10 min). The cell pellet was stored at −80 °C until further use. The frozen cells were re-suspended in HEPES buffer (13 mM, pH 7.0) and diluted to an OD_600_ of 10–15. 7a (1 mM in DMSO, 100 μL) was added to the cell suspension (900 μL) and incubated at 30 °C for 1 h. Samples were centrifuged (4000 rpm, 4 °C). The cell pellet was washed consecutively with DMSO (10%) in HEPES buffer (1×) and HEPES buffer (2×) to remove unbound probe. 1× BugBuster (300 μL, diluted from 10× BugBuster stock with HEPES buffer) was added into each sample. The cell suspension was incubated on a rotating mixer at a slow setting for 20 min at room temperature. Samples were centrifuged (12 000 rpm, 4 °C) for 10 min. The clear supernatant was collected and separated on a 12% tris-glycine SDS-polyacrylamide electrophoresis gel. Upon completion, the gel was washed quickly with pure water. Protein bands were visualised by in-gel fluorescence scanning on a GE Amersham Imager 600, and by Coomassie staining.

## Conclusions

Our results show that pyrazol-3-one 7a can be used as a chemical probe for the fluorescent labelling of the bacterial glycosyltransferase LgtC in recombinant form, cell lysates, and in intact cells. The suitability of pyrazol-3-ones as chemical probes has sometimes been regarded with scepticism.^10^ We show that through judicious choice of the 5-substituent, an appropriate balance of stability and reactivity can be achieved for such applications even in complex mixtures.

To the best of our knowledge, our results represent the first example for the successful labelling of a bacterial glycosyltransferase in intact cells. In contrast to glycosidases, only very few such probes have been reported for glycosyltransferases.^3^ The labelling profile of 7a indicates that the probe is not entirely selective for LgtC, its primary target. However, our insights into the SAR for LgtC inhibition provide a sound basis for further rational optimisation of target selectivity in this new class of chemical probes for glycosyltransferases. Our results suggest that the 7-hydroxycoumarin fluorophore directly contributes to binding at the LgtC active site, most likely by acting as a mimic of the sugar-nucleotide donor. Efforts at further optimisation will be facilitated by our flexible synthetic route, which also allows the rapid variation of the reporter group, including *e.g.*, the introduction of more sensitive commercial fluorophores or pull-down reagents.

Non-catalytic cysteines are a common feature in bacterial glycosyltransferases.^9*b*^ The results from this study therefore also provide an excellent template for the development of similar chemical probes for other bacterial glycosyltransferases. Such probes, including 7a, will be powerful tools to study the role of LgtC and related enzymes for bacterial virulence and pathogenicity.

## Author contributions

G. K. W. conceived and supervised the research and carried out molecular docking experiments; Y. X. performed all other experiments; G. K. W. and Y. X. analysed the data and wrote the manuscript.

## Conflicts of interest

There are no conflicts to declare.

## Supplementary Material

CB-005-D3CB00092C-s001
