# Supplementary material for: A cell-permeable probe for the labelling of a bacterial glycosyltransferase and virulence factor
Source: RSC Chem Biol. 2023 Oct 19;5(1):55–62. doi: 10.1039/d3cb00092c (PMC10763556; doi:10.1039/d3cb00092c)
Supplement: CB-005-D3CB00092C-s001 [file CB-005-D3CB00092C-s001.pdf]

Electronic Supplementary Material (ESI) for RSC Chemical Biology.
This journal is © The Royal Society of Chemistry 2023

Supplementary Information

A cell-permeable probe for the labelling of a bacterial glycosyltransferase and virulence factor

Yong Xu[a] and Gerd K. Wagner*[b]

[a]*Department of Chemistry, King's College London*

[b]*School of Pharmacy, Queen's University Belfast, Medical Biology Centre, 97 Lisburn Road, Belfast BT9 7BL, UK,*

g.wagner@qub.ac.uk

Table of Content

1…. Scheme S1: Synthesis of reporter group azides
2…. Scheme S2: Synthesis of hydrogenated analogue **8**
3…. Fig. S1: Fluorescence emission spectra of **7a**
4…. Fig. S2: Dose-dependent inhibition of LgtC by probe **7a** and inhibitor **1**
5…. Fig. S3: Control experiment with **7a** and CIP
6…. Fig. S4: Molecular docking of **7a** and LgtC
7…. Fig. S5: Bacterial growth control experiment with **7a**
8…. Synthetic protocols
9…. NMR spectra

Scheme S1 Synthesis of reporter group azides[a]

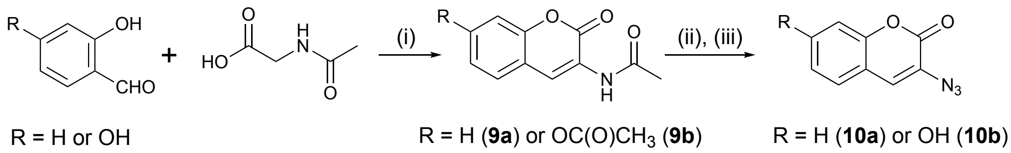

[a]*Reagents & conditions:* (i) Ac_2O, NaOAc, 120 °C, 4h, 20%; (ii) conc. HCl/ethanol (2:1), reflux, 1 h; (iii) $NaNO_2$, then NaN_3, water/ethanol, ice bath. **10a**: 62%, **10b**: 52% (yields over two steps).

Scheme S2 Synthesis of hydrogenated analogue **8**[a]

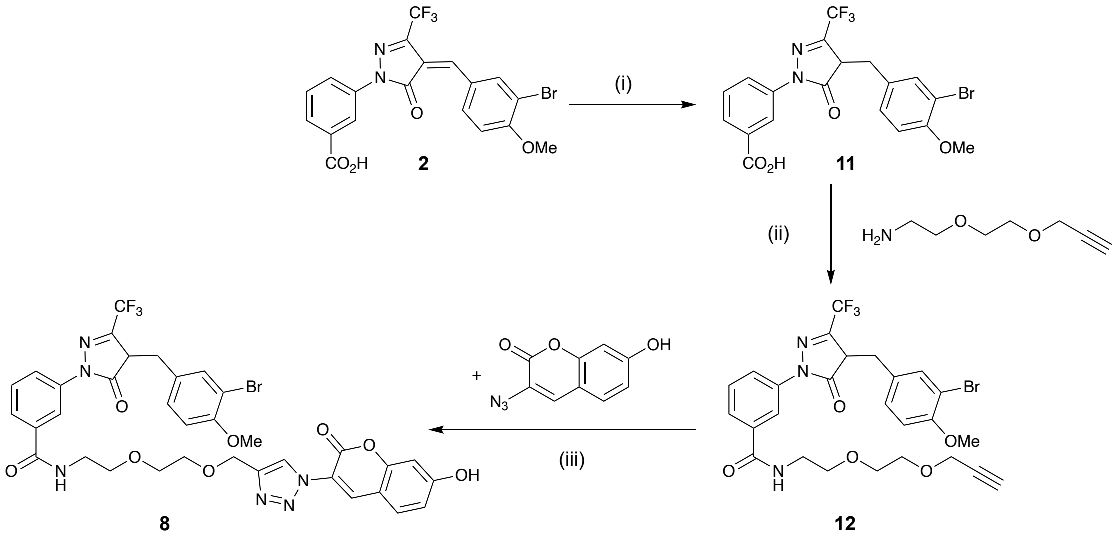

[a]*Reagents & conditions:* (i) $NaBH_4$, aq. dioxane, rt, 30 mins, 88%; (ii) HBTU, DIPEA, DMF, rt, overnight, 78%; (iii) $CuSO_4$, sodium ascorbate, t-BuOH/water (1:1), rt, overnight, 54%.

Figure S1 Fluorescence emission spectra of **7a**[a]

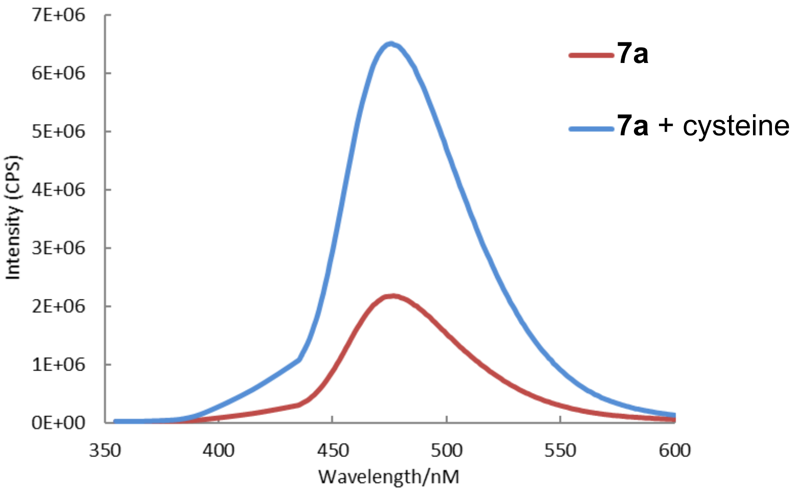

[a]*Conditions*: A solution of **7a** in DMSO (1 mM, 200 μL) was combined with an aqueous solution of L-cysteine (1 mM, 200 μL) or water only (200 μL), followed by dilution with water (1.6 mL). Each solution was stirred at room temperature for 20 min and fluorescence emission was recorded on a Varian Cary Eclipse Spectrometer (λ_{ex} 335 nM).

Figure S2 Dose-dependent inhibition of LgtC by probe **7a** and inhibitor **1**[a]

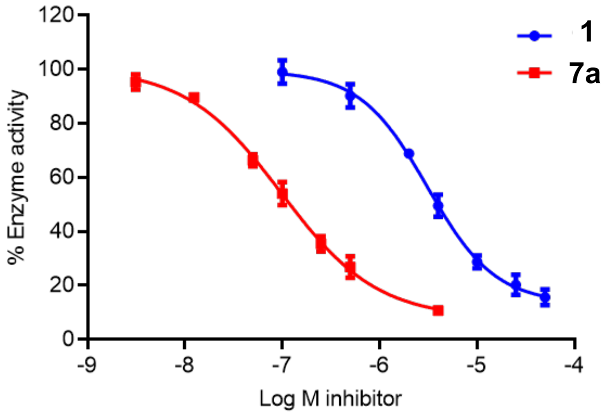

[a]*Conditions*: LgtC was pre-incubated with probe **7a** (0-4.0 μM), inhibitor **1** (0-50 μM) or DMSO, UDP-Gal (28 μM), $MnCl_2$ (5 mM), CIP (10 U/mL), CEL (1 mg/mL), and Triton (0.01%) for 30 mins at 30 °C in HEPES buffer (13 mM, pH 7.0). Lactose (2 mM) was added, and the reactions were incubated for 20 min at 30 °C. Substrate turnover was 32% (**7a**) and 30% (**1**), respectively. Each experiment was carried out in triplicate.

Figure S3 Control experiment with **7a** and CIP[a]

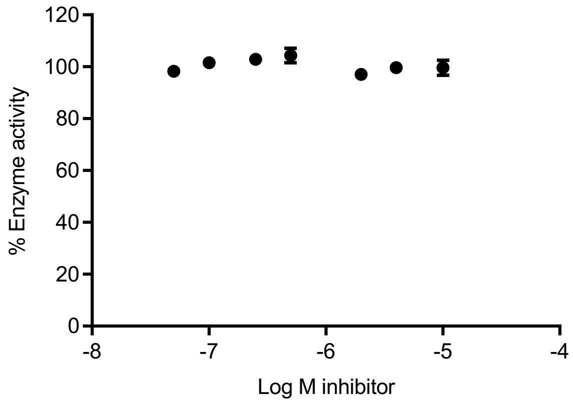

[a]*Conditions*: LgtC, $MnCl_2$ (5 mM), CEL (10 U/mL), Triton (0.01%) and HEPES buffer (13 mM, pH 7.0) were combined with CIP (10 U/mL) or HEPES buffer (13 mM, pH 7.0, control). Probe **7a** at various concentrations (0-10 μM) or DMSO was added. The reaction was started immediately by the addition of UDP (10 μM). Each experiment was carried out in triplicate.

Control experiment with CIP. Compound **7a** was tested for potential inhibition of the calf intestinal alkaline phosphatase (CIP) used in the inhibition assays. CIP was obtained commercially and used as received. The experiment was carried out in Nunc clear, flat-bottom 96-well microplates. All concentrations are final concentrations. HEPES buffer (45 μL, 13 mM, pH 7.0) and aliquots (15 μL) of $MnCl_2$ (5 mM), CEL (1 mg/mL) and Triton X-100 (0.01%) were combined with CIP (15 μL, 10 U/mL) or HEPES buffer (13 mM, pH 7.0, control). **7a** at various concentrations (0-10 μM) in DMSO (15 μL, 10% final DMSO concentration) or DMSO only (15 μL, control) was added. The reaction was started immediately by the addition of UDP (15 μL, 10 μM). Reactions were incubated for 20 mins at 30 °C and stopped by addition of Malachite Green reagent A (30 μL). The microplate was shaken carefully, Malachite Green reagent B (30 μL) was added, and the absorbance was recorded at 620 nm. The absorbance data were used to calculate enzyme activity as previously reported (L. Tedaldi et al., *MedChemCom*m, 2014, **5**, 1193-1201). The experiment was carried out in triplicate.

Figure S4 Molecular docking of **7a** with LgtC

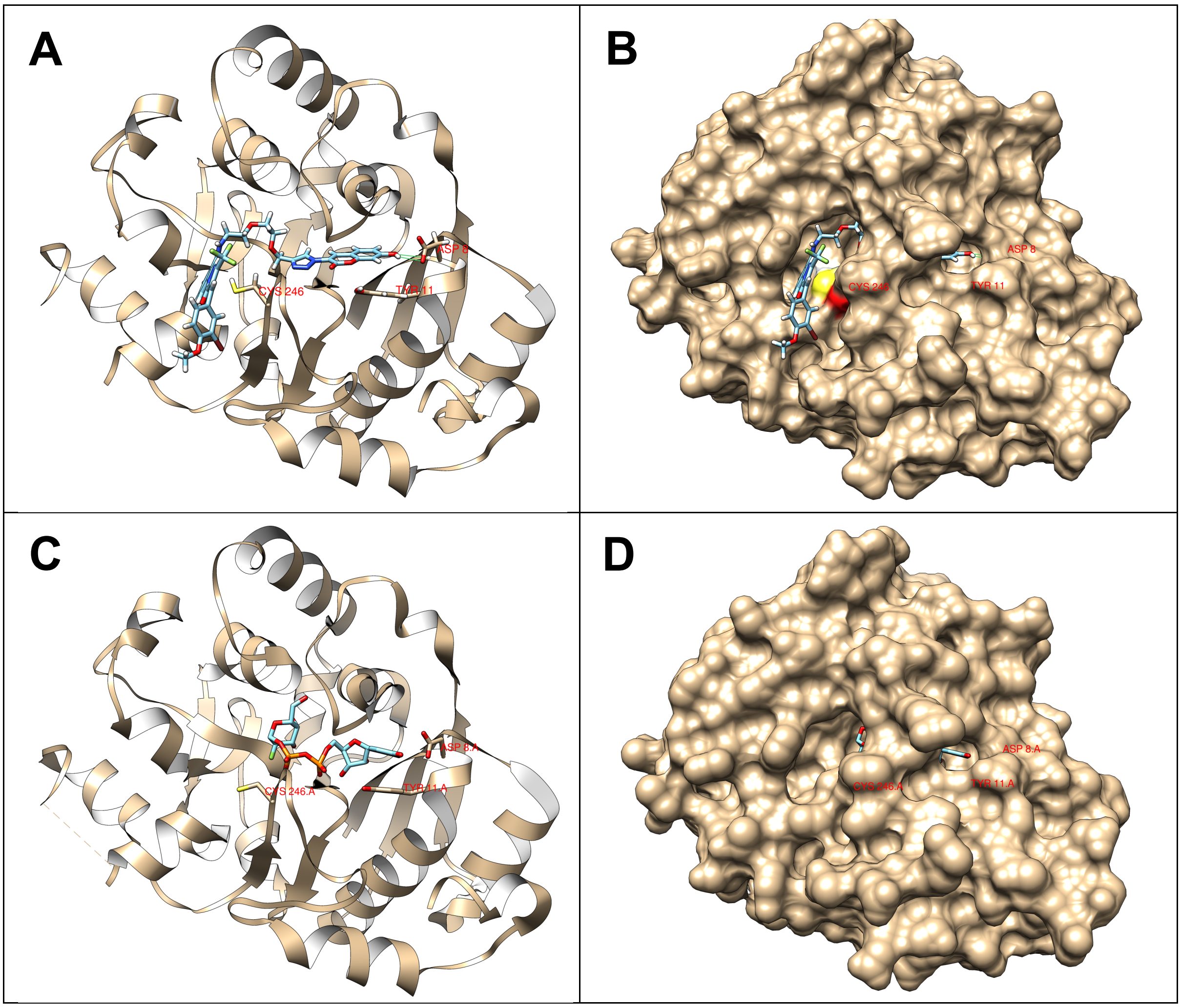

Panels **A** and **B**: Docking pose of **7a** (stick representation, light blue) in the active site of LgtC (PDB 1GA8). The protein (wheat) is shown in cartoon (**A**) or surface (**B**) representation. Panels **C** and **D**: Donor analogue UPF (stick representation, light blue) in complex with LgtC (PDB1GA8). The protein (wheat) is shown in cartoon (**A**) or surface (**B**) representation.

Molecular docking. The ligand input file for **7a** was created in MarvinSketch (version 23.11.0) in .mol2 format and prepared for docking in Chimera (version 1.15). LgtC (PDB 1GA8) was prepared for docking in ChimeraX (version 1.6.1). Docking was performed with SwissDock (http://www.swissdock.ch/) using standard settings and allowing flexibility for side chains within 5Å of any atom of the ligand in its reference binding mode. Docking results were analysed and visualised in Chimera. Fig. S4 shows a representative ligand pose from the lowest energy cluster.

Figure S5 Growth curve of *E. coli* DH5α in the absence or presence of **7a**[a]

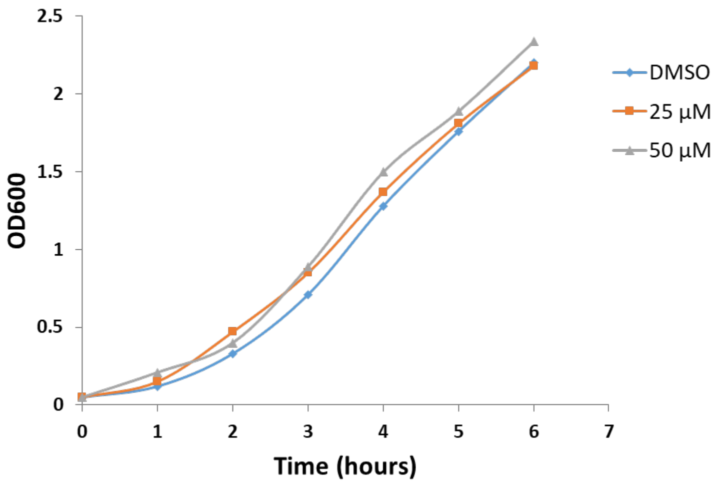

[a]*Conditions*: A single colony was added into LB media and incubated at 37 °C / 180 rpm overnight. The culture was diluted to OD_{600} 0.05 and **7a** (25 µM or 50 µM) or DMSO (control) was added. Cultures were incubated at 37 °C / 180 rpm for 6h and the OD_{600} was recorded at various time points.

Synthetic protocols.

General. All chemical reagents were obtained commercially and used as received. Target compounds and synthetic intermediates were purified by flash chromatography and characterized by TLC, ^{1}H-NMR, ^{13}C-NMR, and ESI-MS. Flash chromatography columns were packed wet. Thin layer chromatography (TLC) was performed on precoated aluminium plates (Silica Gel 60 F254, Merck). Compounds were visualized by exposure to UV light (254/365 nm). NMR spectra were recorded on a Bruker BioSpin at 400 MHz (^{1}H) or 100 MHz (^{13}C). Mass spectra were recorded at the EPSRC National Mass Spectrometry Service Centre, Swansea. Compounds **1**, **2**, **4** and **5** were synthesised as previously reported.[1,2]

***N*-(2-Oxo-2*H*-chromen-3-yl)acetamide (9a).**[3] *N*-Acetylglycine (2.34 g, 20 mmol), salicylaldehyde (2.44 g, 20 mmol), and anhydrous sodium acetate (3.28 g, 40 mmol) were dissolved in acetic anhydride (30 mL) and heated at 120 °C for 4h. The reaction was cooled down to rt and ice-cold water was added. The yellow oily solution was extracted (3x) with ethylacetate. The organic layers were combined and dried over Na_2SO_4. The organic solvent was evaporated to dryness. The residue was triturated with ethylacetate, filtered off, and dried to afford the title compound as a yellow solid in 20% yield (790 mg, 3.9 mmol). **^{1}H-NMR** (DMSO-*d*$_6$,) *δ* (ppm) 2.17 (s, 3H), 7.27-7.37 (m, 1H), 7.39 (d, *J* 8.2 Hz, 1H), 7.45-7.53 (m, 1H), 7.70 (dd, *J* 7.7, 1.5 Hz, 1H), 8.61 (s, 1H), 9.76 (s, 1H). **^{13}C-NMR** (DMSO-*d*$_6$) *δ* (ppm) 23.9, 115.8, 119.5, 123.5, 124.5, 124.9, 127.8, 129.5, 149.6, 157.5, 170.2.

3-Acetamido-2-oxo-2*H*-chromen-7-yl acetate (9b).[4] The title compound was obtained as a yellow solid in 16% yield (789 mg, 3.2 mmol) from *N*-acetylglycine (2.34 g, 20 mmol), 2,4-dihydroxybenzaldehyde (2.76 g, 20 mmol), and anhydrous sodium acetate (4.92 g, 60 mmol) as described for **9a**. **^{1}H-NMR** (DMSO-d_6,): *δ* (ppm) 2.17 (s, 3H), 2.30 (s, 3H), 7.14 (dd, *J* 8.5, 2.2 Hz, 1H), 7.28 (d, *J* 2.1 Hz, 1H), 7.76 (d, *J* 8.5 Hz, 1H), 8.63 (s, 1H), 9.78 (s, 1H).

3-Azido-2*H*-chromen-2-one (10a).[3] **9a** (609 mg, 3 mmol) was dissolved in conc. HCl/ethanol (2:1) and heated to reflux for 1h. The reaction was cooled to room temperature, diluted with ice water, and placed in an ice bath. Sodium nitrite (414 mg, 6.0 mmol) was added, and the reaction was stirred for 10 mins. Sodium azide (594 mg, 9.0 mmol) was added in portions, and the solution was stirred for another 15 mins. The yellow precipitate was filtered off, washed with cold water, and dried under reduced pressure to afford the final compound as a yellow solid in 62% yield (350 mg, 1.86 mmol). **^{1}H-NMR** ($CDCl_3$): *δ* (ppm) 7.23 (s, 1H), 7.29-7.34 (m, 1H), 7.34-7.39 (m, 1H), 7.43-7.46 (m, 1H), 7.46-7.52 (m, 1H). **^{13}C-NMR** ($CDCl_3$): *δ* (ppm) 116.5, 119.3, 125.1, 125.7, 126.5, 127.3, 130.4, 151.3, 157.5.

3-Azido-7-hydroxy-2*H*-chromen-2-one (10b).[4] The title compound was obtained from **9b** (675 mg, 2.6 mmol), sodium nitrite (534 mg, 7.7 mmol), and sodium azide (754 mg, 11.6 mmol) in 52% yield (274 mg, 1.34 mmol) after column chromatography, as described for **10a**. **^{1}H-NMR** (DMSO-d_6): *δ* (ppm) 6.76 (d, *J* 2.3 Hz, 1H), 6.81 (dd, *J* 8.5, 2.3 Hz, 1H), 7.48 (d, *J* 8.5 Hz, 1H), 7.60 (s, 1H), 10.54 (s, 1H). **^{13}C-NMR** (DMSO-d_6): *δ* (ppm) 102.0, 111.3, 113.8, 121.1, 127.8, 129.1, 152.7, 157.3, 160.2.

***N*-(2-(2-((1-(7-Hydroxy-2-oxo-2*H*-chromen-3-yl)-1*H*-1,2,3-triazol-4-yl)methoxy)ethoxy)ethyl)-3-(5-hydroxy-3-(trifluoromethyl)-1*H*-pyrazol-1-yl)benzamide (6a)**. **5b** (119 mg, 0.3 mmol) and **10b** (61 mg, 0.3 mmol) were suspended in water/t-BuOH (1:1, 10 mL). Aq. sodium ascorbate (1M, 60 µL) was added, followed by aq. copper (II) sulfate pentahydrate (0.3M, 20 µL). The mixture was stirred in the dark at rt overnight. Upon completion, all solvents were evaporated. The residue was purified by flash column chromatography to afford the title compound as a light yellow solid in 30% yield (75 mg, 0.09 mmol). **^{1}H-NMR** (DMSO-d_6): *δ* (ppm) 3.40-3.48 (m, 2H), 3.56 (t, *J* 5.9 Hz, 2H), 3.58-3.67 (m, 4H), 4.63 (s, 2H), 5.95 (s, 1H), 6.85 (d, *J* 2.1 Hz, 1H), 6.91 (dd, *J* 8.5, 2.2 Hz, 1H), 7.59 (t, *J* 7.9 Hz, 1H), 7.74 (d, *J* 8.6 Hz, 1H), 7.80-7.90 (m, 2H), 8.17 (s, 1H), 8.51 (s, 1H), 8.59 (s, 1H), 8.72 (t, *J* 5.4 Hz, 1H), 10.91 (s, 1H), 12.61 (s, 1H).

***N*-(2-((1-(7-Hydroxy-2-oxo-2*H*-chromen-3-yl)-1H-1,2,3-triazol-4-yl)methoxy)ethyl)-3-(5-hydroxy-3-(trifluoromethyl)-1*H*-pyrazol-1-yl)benzamide (6b)**. The title compound was obtained as a light yellow solid in 44% yield (120 mg, 0.22 mmol) from **5a** (176 mg, 0.5 mmol) and **10b** (102 mg, 0.5 mmol) via the method described for **6a**. **^{1}H-NMR** (DMSO-d_6): *δ* (ppm)

3.42-3.51 (m, 2H), 3.64 (t, *J* 5.9 Hz, 2H), 4.66 (s, 2H), 5.93 (s, 1H), 6.85 (d, *J* 2.1 Hz, 1H), 6.91 (dd, *J* 8.5, 2.2 Hz, 1H), 7.59 (t, *J* 7.9 Hz, 1H), 7.73 (d, *J* 8.6 Hz, 1H), 7.86 (t, *J* 8.2 Hz, 2H), 8.19 (s, 1H), 8.56 (d, *J* 7.3 Hz, 2H), 8.76 (t, *J* 5.5 Hz, 1H), 10.92 (s, 1H), 12.69 (s, 1H).

3-(5-Hydroxy-3-(trifluoromethyl)-1*H*-pyrazol-1-yl)-*N*-(2-((1-phenyl-1H-1,2,3-triazol-4-yl)methoxy)ethyl)benzamide (6c). The title compound was obtained as a light yellow solid in 63% yield (293 mg, 0.63 mmol) from **4a** (353 mg, 1.0 mmol) and phenylazide[5] (119 mg, 1.0 mmol) via the method described for **6a**. **^{1}H-NMR** (d_3-MeOD): δ (ppm) 3.53 (t, *J* 5.4 Hz, 2H), 3.67 (t, *J* 5.4 Hz, 2H), 4.64 (s, 2H), 7.33-7.40 (m, 1H), 7.40-7.48 (m, 3H), 7.63-7.72 (m, 3H), 7.78-7.85 (m, 1H), 8.12 (t, *J* 1.9 Hz, 1H), 8.39 (s, 1H).

3-(5-Hydroxy-3-methyl-1*H*-pyrazol-1-yl)-*N*-(2-(2-((1-(2-oxo-2*H*-chromen-3-yl)-1*H*-1,2,3-triazol-4-yl)methoxy)ethoxy)ethyl)benzamide (6d). The title compound was obtained as a light yellow solid in 68% yield (350 mg, 0.6 mmol) from **5b** (350 mg, 0.88 mmol) and **10a** (165 mg, 0.88 mmol) via the method described for **6a**. **^{1}H-NMR** (DMSO-d_6): δ (ppm) 3.71-3.79 (m, 6H), 3.79-3.86 (m, 2H), 4.81 (s, 2H), 6.07 (s, 1H), 7.16 (s, 1H), 7.44-7.51 (m, 2H), 7.55 (t, *J* 8.0 Hz, 1H), 7.68-7.74 (m, 1H), 7.76 (dd, *J* 7.7, 1.3 Hz, 1H), 7.85 (d, *J* 7.8 Hz, 1H), 8.02 (d, *J* 8.9 Hz, 1H), 8.41 (s, 1H), 8.61 (s, 1H), 8.77 (s, 1H).

(Z)-3-(4-(3-Bromo-4-methoxybenzylidene)-5-oxo-3-(trifluoromethyl)-4,5-dihydro-1*H*-pyrazol-1-yl)-*N*-(2-(2-((1-(7-hydroxy-2-oxo-2*H*-chromen-3-yl)-1*H*-1,2,3-triazol-4-yl)methoxy)ethoxy)ethyl)benzamide (7a). **6a** (60 mg, 0.1 mmol) and 3-bromo-4-methoxybenzaldehyde (33 mg, 0.15 mmol) were placed in a microwave-proof glass tube and heated for 15 min at 160 °C in a commercial microwave apparatus. The reaction was cooled to room temperature. The reaction product was precipitated by addition of ethyl acetate and hexane, collected by filtration, and recrystallized from hexane (5 mL) and ethyl acetate (0.5 mL). The title compound was obtained as an orange solid in 31% yield (25 mg, 0.03 mmol). **^{1}H-NMR** (DMSO-d_6, ppm): δ (ppm) 3.44-3.49 (m, 2H), 3.54-3.68 (m, 6H), 4.03 (s, 3H), 4.63 (s, 2H), 6.81 (d, *J* 2.1 Hz, 1H), 6.88 (dd, *J* 8.5, 2.2 Hz, 1H), 7.35 (d, *J* 8.9 Hz, 1H), 7.57 (t, *J* 7.9 Hz, 1H), 7.71 (d, *J* 8.6 Hz, 1H), 7.78 (d, *J* 8.0 Hz, 1H), 7.90 (s, 1H), 7.94-8.00 (m, 1H), 8.28 (t, *J* 1.8 Hz, 1H), 8.46 (s, 1H), 8.54 (s, 1H), 8.66 (m, 2H), 9.28 (d, *J* 2.1 Hz, 1H), 10.88 (s, 1H). **^{13}C-NMR** (DMSO-d_6): δ (ppm) 39.3, 57.1, 63.2, 68.8, 69.0, 69.6, 102.1, 110.3, 111.0, 112.8, 114.2, 118.7, 118.9, 119.2, 119.7 (q, $^1J_{CF}$ 270 Hz), 122.4, 124.6, 124.7, 126.7, 129.0, 130.9, 135.3, 136.2, 137.2, 138.0, 138.9, 139.9 (d, $^2J_{CF}$ 37 Hz), 144.1, 148.8, 154.6, 156.2, 160.7, 161.1, 162.4, 165.6. **ESI-MS**: m/z 797.1 (35%) $[M+H]^+$, 819.1 (100%) $[M+Na]^+$, 851.1 (30%) $[M+MeOH+Na]^+$; **HR-MS**: m/z 797.1177 $[M+H]^+$, $[C_{35}H_{29}O_8N_6BrF_3]^+$ requires 797.1177.

(Z)-3-(4-(3-Bromo-4-methoxybenzylidene)-5-oxo-3-(trifluoromethyl)-4,5-dihydro-1*H*-pyrazol-1-yl)-*N*-(2-((1-(7-hydroxy-2-oxo-2*H*-chromen-3-yl)-1H-1,2,3-triazol-4-

yl)methoxy)ethyl)benzamide (7b). The title compound was obtained as an orange solid in 71% yield (53 mg, 0.07 mmol) from **6b** (53 mg, 0.1 mmol) and 3-bromo-4-methoxybenzaldehyde (33 mg, 0.15 mmol) via the method described for **7a**. **^{1}H-NMR** (DMSO-*d*$_6$): *δ* (ppm) 3.46-3.52 (m, 2H), 3.66 (t, *J* 5.8 Hz, 2H), 4.03 (s, 3H), 4.67 (s, 2H), 6.81 (d, *J* 2.1 Hz, 1H), 6.87 (dd, *J* 8.6, 2.2 Hz, 1H), 7.37 (d, *J* 8.9 Hz, 1H), 7.59 (t, *J* 8.0 Hz, 1H), 7.70 (d, *J* 8.6 Hz, 1H), 7.80 (d, *J* 7.8 Hz, 1H), 7.92 (s, 1H), 7.98 (dd, *J* 7.7, 1.8 Hz, 1H), 8.30 (s, 1H), 8.53 (d, *J* 5.1 Hz, 2H), 8.69 (dd, *J* 8.8, 2.0 Hz, 1H), 8.74 (t, *J* 5.5 Hz, 1H), 9.31 (d, *J* 2.0 Hz, 1H), 10.89 (s, 1H). **^{13}C-NMR** (DMSO-*d*$_6$): *δ* (ppm) 39.1, 57.0, 63.2, 68.2, 102.0, 110.1, 111.1, 112.5, 114.2, 118.5, 118.7, 119.0, 122.0, 124.5, 126.7, 128.8, 130.7, 135.2, 135.8, 137.2, 137.9, 139.0, 140.3, 144.1, 148.8, 154.5, 156.1, 160.7, 161.0, 162.4, 165.6. **ESI-MS**: m/z 753.1 (40%) $[M+H]^+$, 785.1 (80%) $[M+MeOH+H]^+$, 807.1 (30%) $[M+MeOH+Na]^+$; **HR-MS**: m/z 753.0914 $[M+H]^+$, $[C_{33}H_{25}O_7N_6BrF_3]^+$ requires 753.0915.

(Z)-3-(4-(3-Bromo-4-methoxybenzylidene)-5-oxo-3-(trifluoromethyl)-4,5-dihydro-1*H*-pyrazol-1-yl)-*N*-(2-((1-phenyl-1*H*-1,2,3-triazol-4-yl)methoxy)ethyl)benzamide (7c). The title compound was obtained as an orange solid in 60% yield (80 mg, 0.12 mmol) from **6c** (95 mg, 0.2 mmol) and 3-bromo-4-methoxybenzaldehyde (65 mg, 0.3 mmol) via the method described for **7a**. **^{1}H-NMR** ($CDCl_3$): *δ* (ppm) 3.61-3.71 (m, 2H), 3.75 (t, *J* 4.7 Hz, 2H), 3.95 (s, 3H), 4.73 (s, 2H), 6.77 (s, 1H), 6.94 (d, *J* 8.8 Hz, 1H), 7.29-7.47 (m, 4H), 7.53 (s, 1H), 7.58 (d, *J* 8.0 Hz, 2H), 7.67 (d, *J* 7.6 Hz, 1H), 7.96 (s, 1H), 8.00 (d, *J* 8.1 Hz, 1H), 8.30 (s, 1H), 8.59 (d, *J* 8.7 Hz, 1H), 8.74 (s, 1H). **^{13}C-NMR** ($CDCl_3$): *δ* (ppm) 40.0, 56.8, 64.4, 69.3, 111.7, 112.3, 117.9, 119.4, 119.8 (q, $^1J_{CF}$ 270 Hz), 120.6, 120.8, 122.5, 125.0, 126.9, 128.8, 129.4, 129.7, 135.4, 136.8, 137.0, 137.8, 140.0, 141.0 (d, $^2J_{CF}$ 37 Hz), 145.5, 148.7, 161.1, 166.9. **ESI-MS**: m/z 669.1 (38%) $[M+H]^+$, 701.1 (100%) $[M+MeOH+H]^+$, 723.1 (15%) $[M+MeOH+Na]^+$; **HR-MS**: m/z 669.1066 $[M+H]^+$, $[C_{30}H_{25}O_4N_6BrF_3]^+$ requires 669.1067.

(Z)-3-(4-(3-Bromo-4-methoxybenzylidene)-5-oxo-3-(trifluoromethyl)-4,5-dihydro-1*H*-pyrazol-1-yl)-*N*-(2-(2-((1-(2-oxo-2*H*-chromen-3-yl)-1*H*-1,2,3-triazol-4-yl)methoxy)ethoxy)ethyl)benzamide (7d). The title compound was obtained as an orange solid in 47% yield (60 mg, 0.07 mmol) from **6d** (88 mg, 0.15 mmol) and 3-bromo-4-methoxybenzaldehyde (50 mg, 0.23 mmol) via the method described for **7a**. **^{1}H-NMR** ($CDCl_3$): *δ* (ppm) 3.71-3.77 (m, 6H), 3.79-3.84 (m, 2H), 4.06 (s, 3H), 4.82 (s, 2H), 7.02 (d, *J* 8.8 Hz, 1H), 7.22 (s, 1H), 7.36-7.43 (m, 2H), 7.50 (t, *J* 8.0 Hz, 1H), 7.54 (s, 1H), 7.58-7.65 (m, 2H), 7.77 (d, *J* 7.9 Hz, 1H), 8.04 (d, *J* 9.3 Hz, 1H), 8.37 (t, *J* 1.8 Hz, 1H), 8.47 (s, 1H), 8.64-8.70 (m, 2H), 8.75 (d, *J* 2.2 Hz, 1H). **^{13}C-NMR** ($CDCl_3$): *δ* (ppm) 39.9, 56.8, 64.4, 69.7, 70.0, 70.4, 111.7, 112.2, 116.7, 118.0, 118.2, 119.4, 119.7 (q, $^1J_{CF}$ 270 Hz), 122.3, 122.9, 123.7, 125.1, 125.5, 126.8, 128.9, 129.2, 132.7, 132.9, 135.6, 136.7, 137.6, 134.0, 140.8 (d, $^2J_{CF}$ 37 Hz), 145.4, 148.3, 152.5, 155.8, 161.0, 161.3, 166.9. ESI-MS: m/z 781.1 (40%) $[M+H]^+$, 813.1 (100%)

[M+MeOH+H]$^+$, 835.1 (45%) [M+MeOH+Na]$^+$; HR-MS: m/z 781.1227 [M+H]$^+$, $[C_{35}H_{29}O_7N_6BrF_3]^+$ requires 781.1228.

3-(4-(3-Bromo-4-methoxybenzyl)-5-hydroxy-3-(trifluoromethyl)-1*H*-pyrazol-1-yl)benzoic acid (11). A solution of **2**[6] (147 mg, 0.31 mmol) in dioxane was added dropwise to a stirred solution of sodium borohydride in water/dioxane (1:1). After addition was complete, stirring was continued for 30 mins at room temperature. Upon completion, the organic solvent was evaporated, and the pH of the aqueous solution was adjusted to 3 with dilute hydrochloric acid. The yellow precipitate was filtered off and washed with cold water to afford the title compound in 88% yield (129 mg, 0.27 mmol). **^{1}H-NMR** (DMSO): *δ* (ppm) 3.80 (s, 3H), 3.81 (s, 2H), 7.02 (d, *J* 8.5 Hz, 1H), 7.13 (dd, *J* 8.5, 2.1 Hz, 1H), 7.36 (d, *J* 2.1 Hz, 1H), 7.65 (t, *J* 8.0 Hz, 1H), 7.91-7.95 (m, 1H), 8.00-8.07 (m, 1H), 8.33 (t, *J* 1.8 Hz, 1H).

3-(4-(3-Bromo-4-methoxybenzyl)-5-hydroxy-3-(trifluoromethyl)-1*H*-pyrazol-1-yl)-*N*-(2-(2-(prop-2-yn-1-yloxy)ethoxy)ethyl)benzamide (12). **11** (129 mg, 0.27 mmol), HBTU (112 mg, 0.30 mmol), and DIPEA (70 mg, 0.54 mmol) were dissolved in DMF, followed by addition of 2-(2-(prop-2-yn-1-yloxy)ethoxy)ethan-1-amine (60 mg, 0.41 mmol). The reaction mixture was stirred at rt for 16 hours and evaporated to dryness. The residue was purified by flash column chromatography to afford the title compound as a light yellow solid in 78% yield (123 mg, 0.21 mmol). **^{1}H-NMR** ($CDCl_3$): *δ* (ppm) 2.77 (s, 1H), 2.85 (s, 2H), 3.51-3.56 (m, 2H), 3.66-3.70 (m, 4H), 3.82 (s, 3H), 3.83 (s, 2H), 4.15-4.17 (m, 2H), 6.70 (d, *J* 8.5 Hz, 1H), 7.02 (dd, *J* 8.4, 2.1 Hz, 1H), 7.13 (t, *J* 5.3 Hz, 1H), 7.35 (d, *J* 2.1 Hz, 1H), 7.43 (t, *J* 7.9 Hz, 1H), 7.53 (d, *J* 7.9 Hz, 1H), 7.87 (s, 1H), 7.92 (m, 1H), 8.22 (t, *J* 1.8 Hz, 1H).

3-(4-(3-Bromo-4-methoxybenzyl)-5-hydroxy-3-(trifluoromethyl)-1*H*-pyrazol-1-yl)-*N*-(2-(2-((1-(7-hydroxy-2-oxo-2*H*-chromen-3-yl)-1*H*-1,2,3-triazol-4-yl)methoxy)ethoxy)ethyl)benzamide (8). The title compound was obtained as a light yellow solid in 54% yield (55 mg, 0.07 mmol) from **12** (80 mg, 0.13 mmol) and **10b** (27 mg, 0.13 mmol) via the method described for **7a**. **^{1}H-NMR** (*d*$_3$-MeOD): *δ* (ppm) 3.49 (t, *J* 5.2 Hz, 2H), 3.55-3.61 (m, 4H), 3.61-3.64 (m, 2H), 3.68 (s, 2H), 3.71 (s, 3H), 4.60 (s, 2H), 6.68 (d, *J* 2.1 Hz, 1H), 6.77 (dd, *J* 8.6, 2.2 Hz, 1H), 6.80 (d, *J* 8.5 Hz, 1H), 6.98 (dd, *J* 8.4, 1.7 Hz, 1H), 7.22 (d, *J* 1.8 Hz, 1H), 7.40-7.50 (m, 2H), 7.69 (d, *J* 7.9 Hz, 1H), 7.76 (d, *J* 8.0 Hz, 1H), 8.09 (s, 1H), 8.27 (s, 1H), 8.38 (s, 1H). **^{13}C-NMR** (*d*$_3$-MeOD): *δ* (ppm) 26.9, 41.1, 56.7, 64.9, 70.5, 70.9, 71.3, 112.3, 111.9, 103.4, 101.3, 113.2, 115.6, 120.7, 122.8, 123.1 (q, $^1J_{CF}$ 270 Hz), 125.9, 126.7, 127.1, 129.1, 130.4, 131.9, 133.7, 135.1, 136.7, 137.1, 139.7, 141.4 (d, $^2J_{CF}$ 37 Hz), 145.9, 153.0, 155.8, 156.5, 158.1, 164.3, 169.2. **ESI-MS**: m/z 799.1 (100%) [M+H]$^+$, 821.1 (35%) [M+Na]$^+$; **HR-MS**: m/z 799.1325 [M+H]$^+$, $[C_{35}H_{31}BrF_3N_6O_8]^+$ requires 799.1333.

References

1 Y. Xu, N. Uddin and G. K. Wagner, Chapter 9: Covalent Probes for Carbohydrate-Active Enzymes: From Glycosidases to Glycosyltransferases. In: *Methods in Enzymology* (Imperiali, B., Ed.), 2018, pp 237-265, Academic Press.

2 Y. Xu, R. Smith, M. Vivoli, M. Ema, N. Göös, S. Gehrke, N. J. Harmer and G. K. Wagner, *Biorg. Med. Chem.*, 2017, **25**, 3182-3194.

3 R. Rajaganesh, P. Ravinder, V. Subramanian and T. Mohan Das, *Carbohydr. Res.*, 2011, **346**, 2327-2336.

4 W. Li, W. Sun, X. Yu, L. Du and M. Li, *J. Fluoresc.*, 2013, **23**, 181-186.

5 G. Colombano, C. Albani, G. Ottonello, A. Ribeiro, R. Scarpelli, G. Tarozzo, J. Daglian, K.-M. Jung, D. Piomelli and T. Bandiera, *ChemMedChem*, 2015, **10**, 380-395.

6 Y. Xu, J. Cuccui, C. Denman, T. Maharjan, B. W. Wren and G. K. Wagner, *Biorg. Med. Chem.*, 2018, **26**, 2973-2983.

^{1}H and ^{13}C NMR spectra for 7a-d and 8 (final compounds), and 6a-d, 9a, 9b, 10a and 10b (intermediates

<u>7a</u>

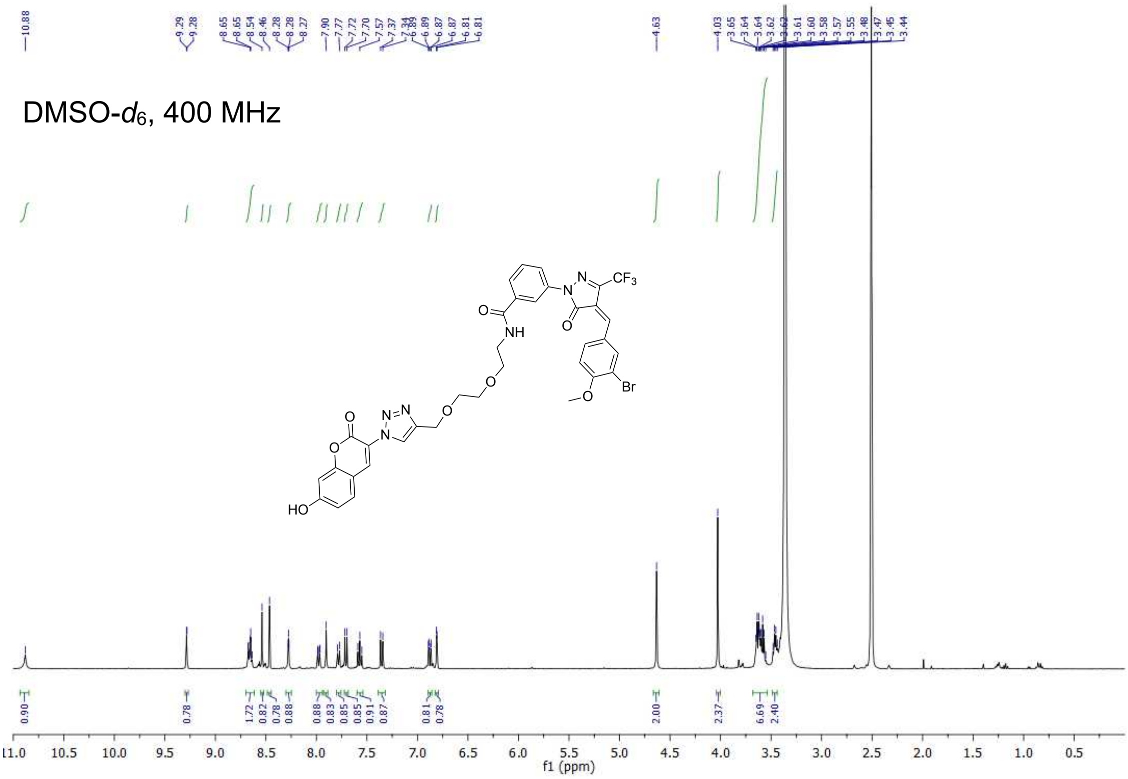

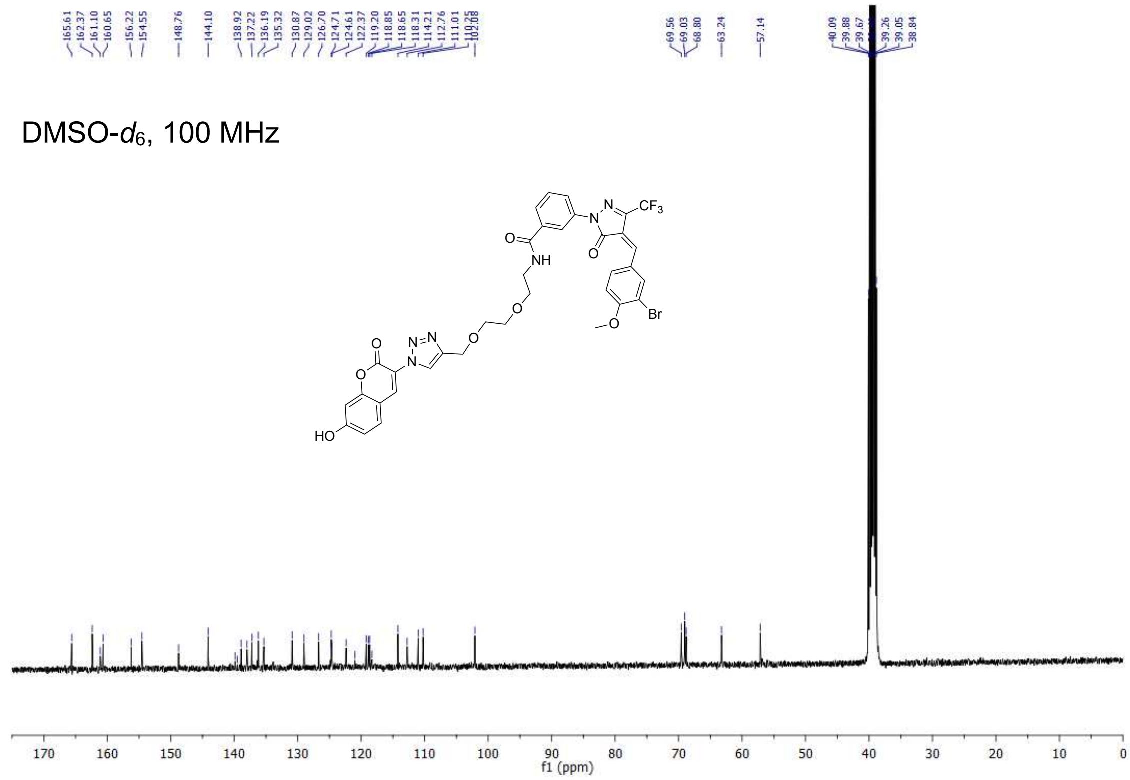

7b

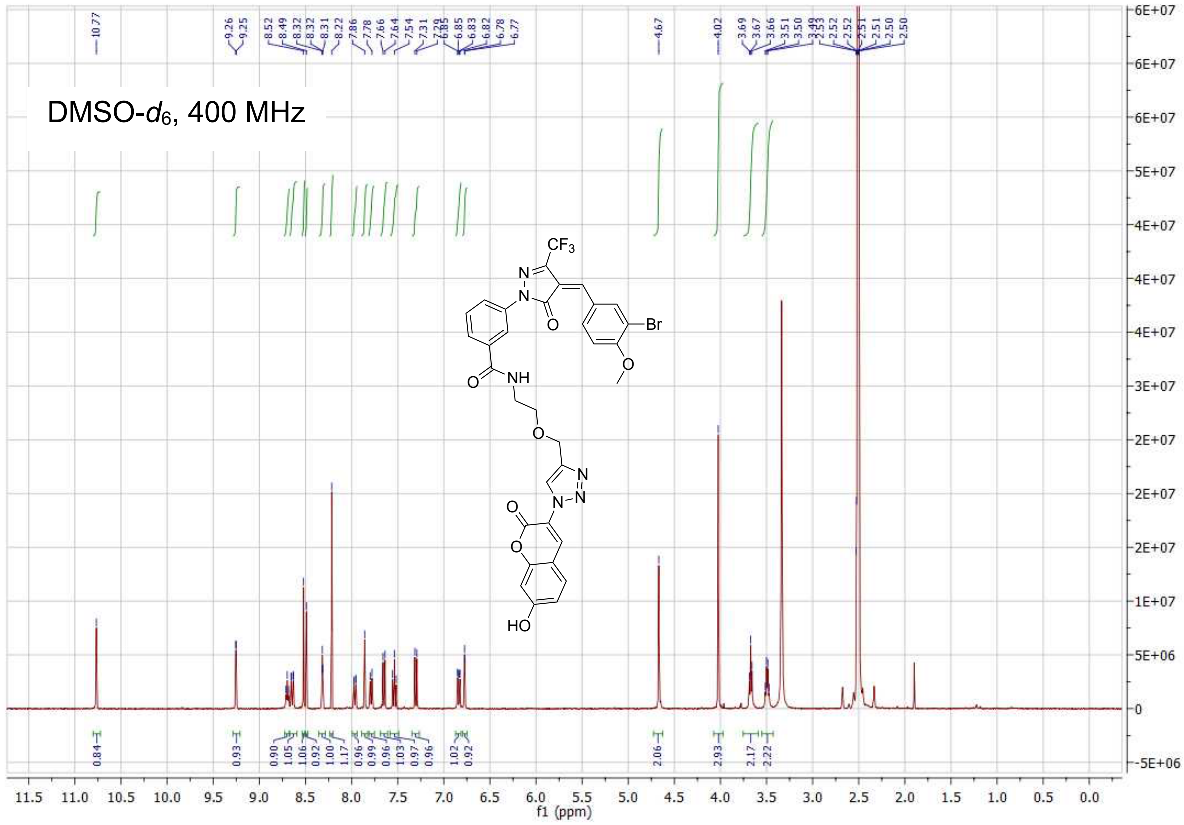
DMSO-d_6, 400 MHz
CF3
N
N
O
Br
O
NH
O
O
N
N
N
O
O
HO
f1 (ppm)

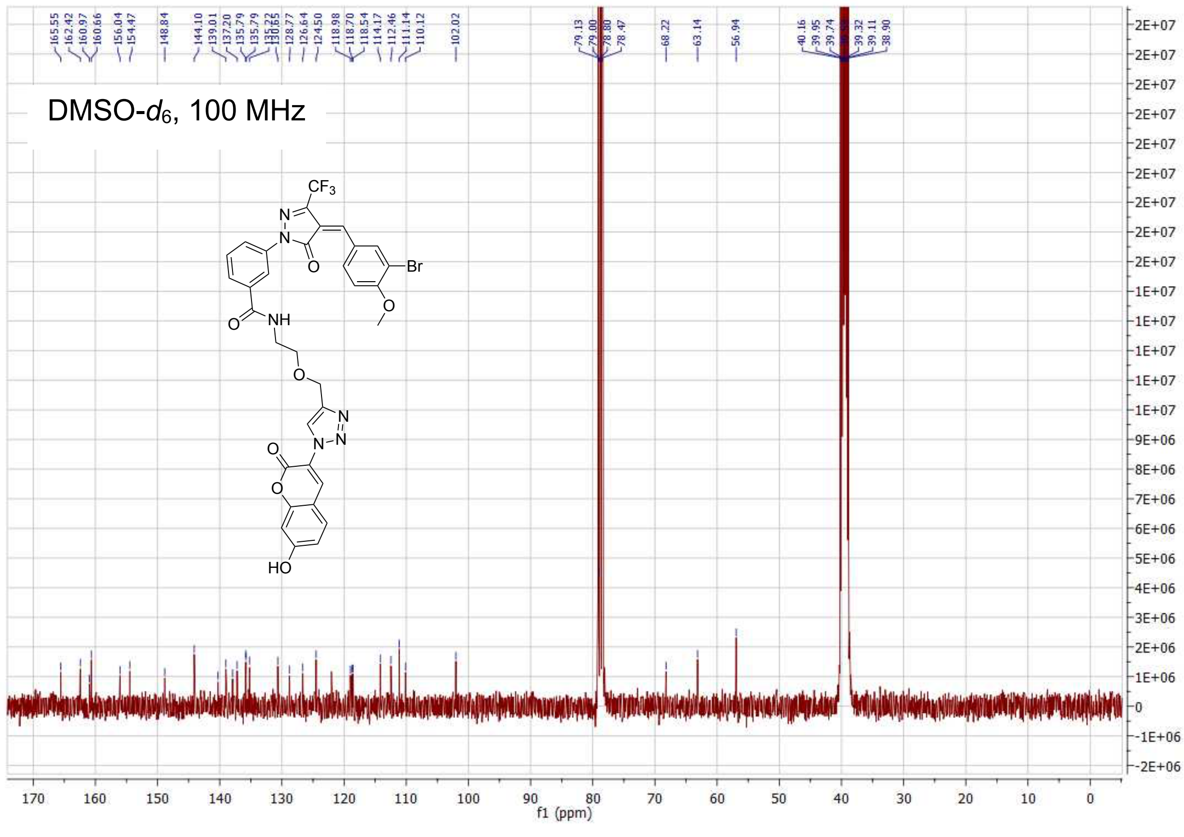
DMSO-d_6, 100 MHz
CF3
N
N
O
Br
O
NH
O
O
N
N
N
O
O
HO
f1 (ppm)

7c

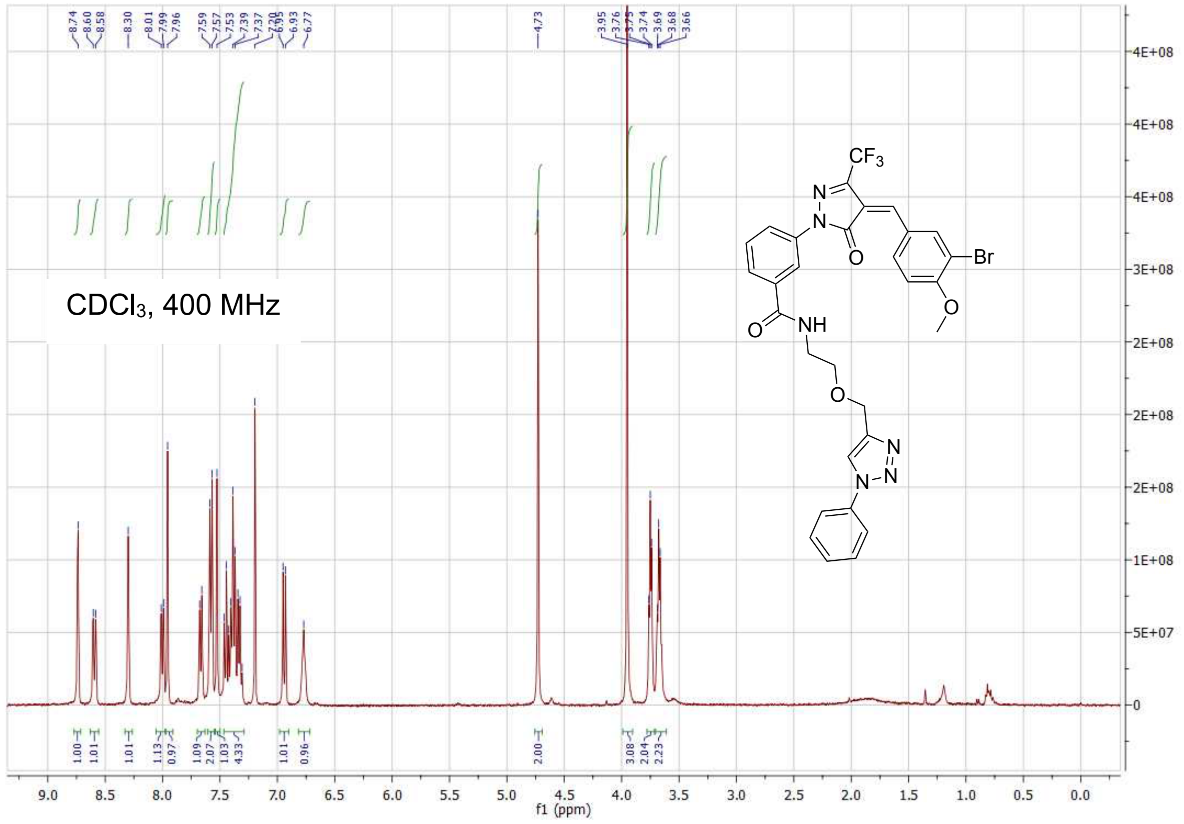

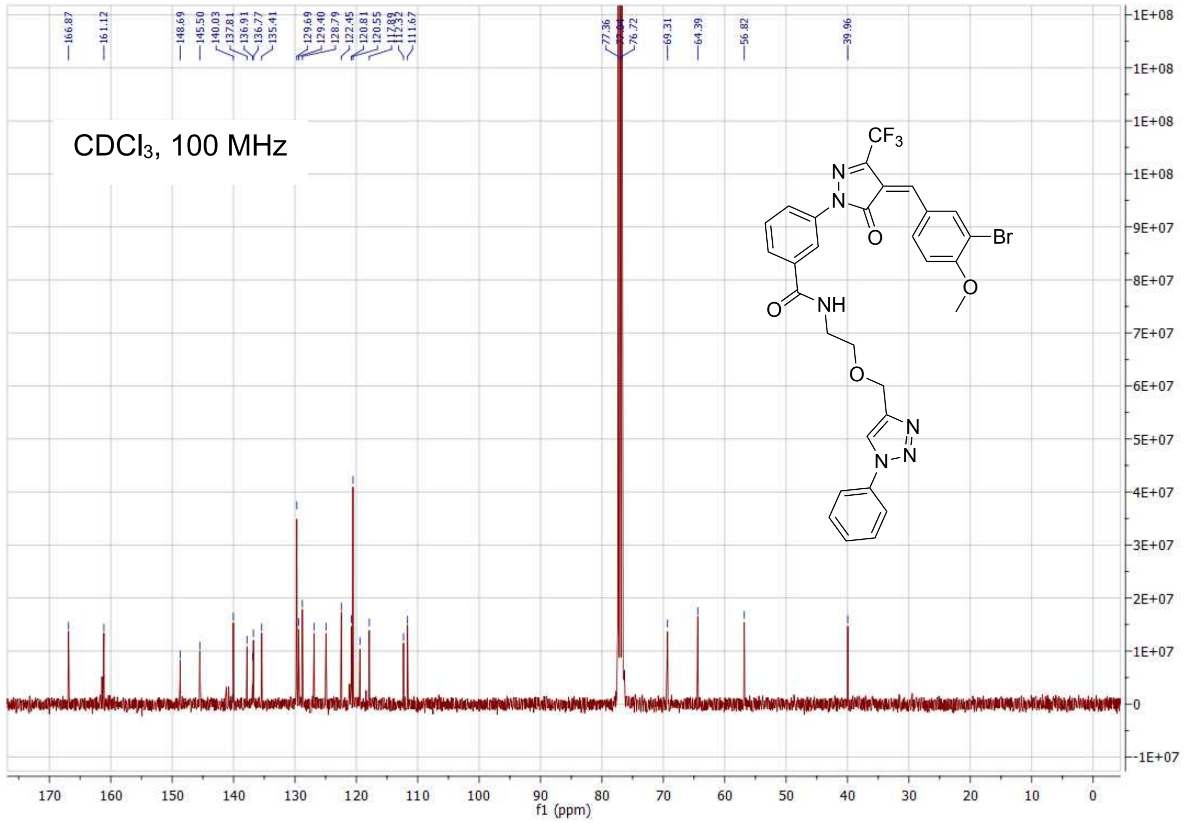

7d

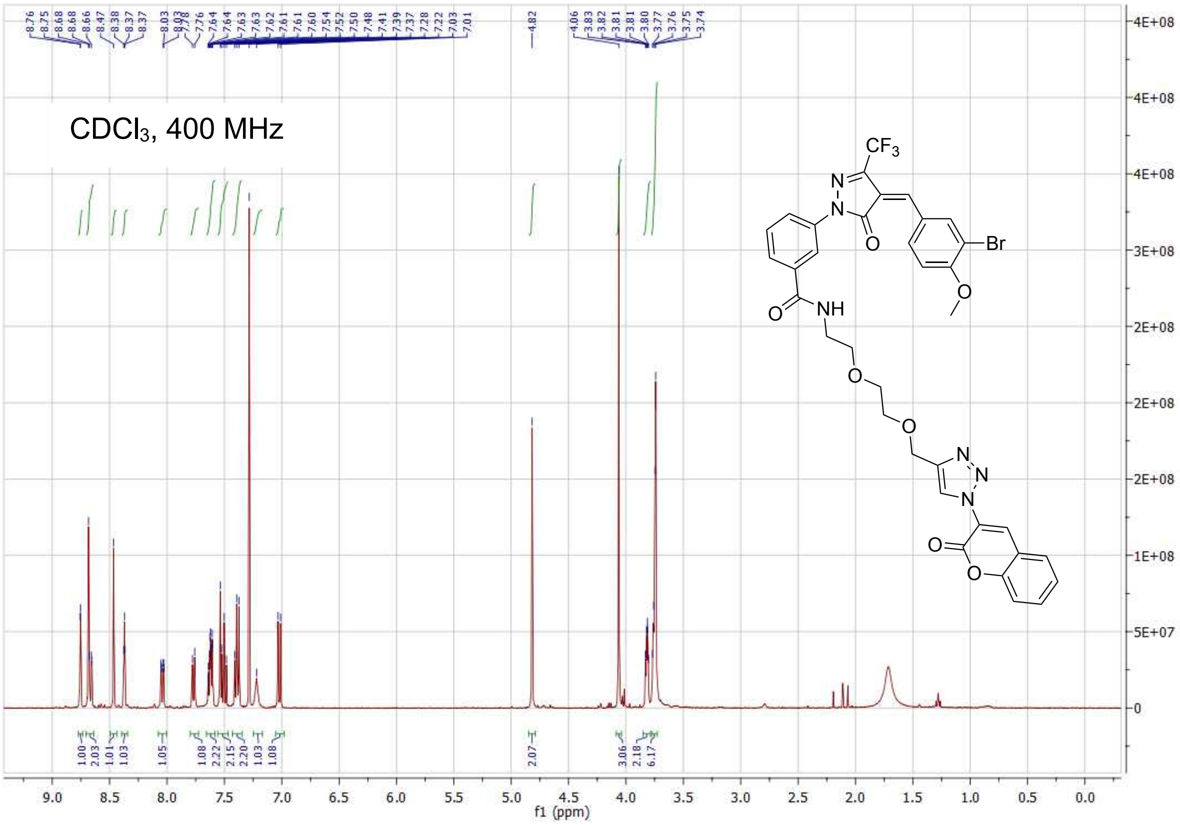
CDCl3, 400 MHz
CF3
N
N
O
Br
O
NH
O
O
N
N
N
O
O
f1 (ppm)

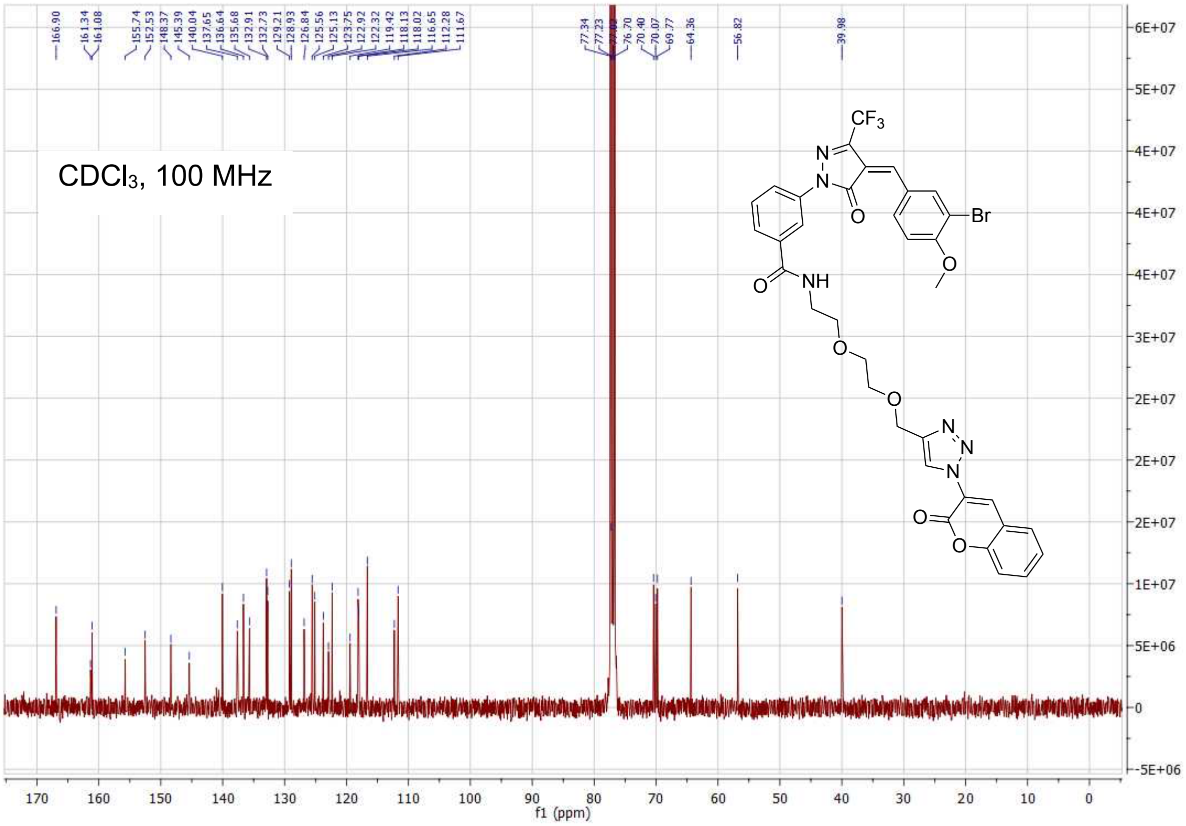
CDCl3, 100 MHz
CF3
Br
NH
f1 (ppm)

8

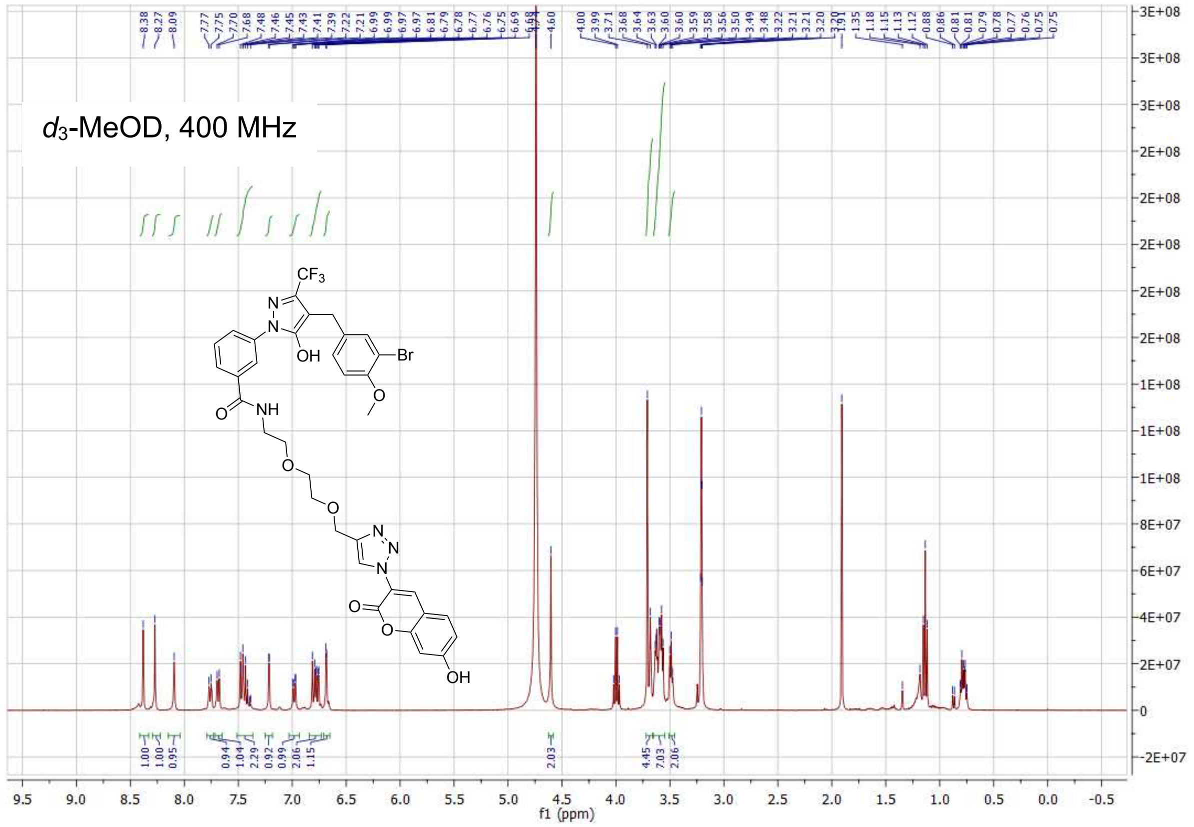

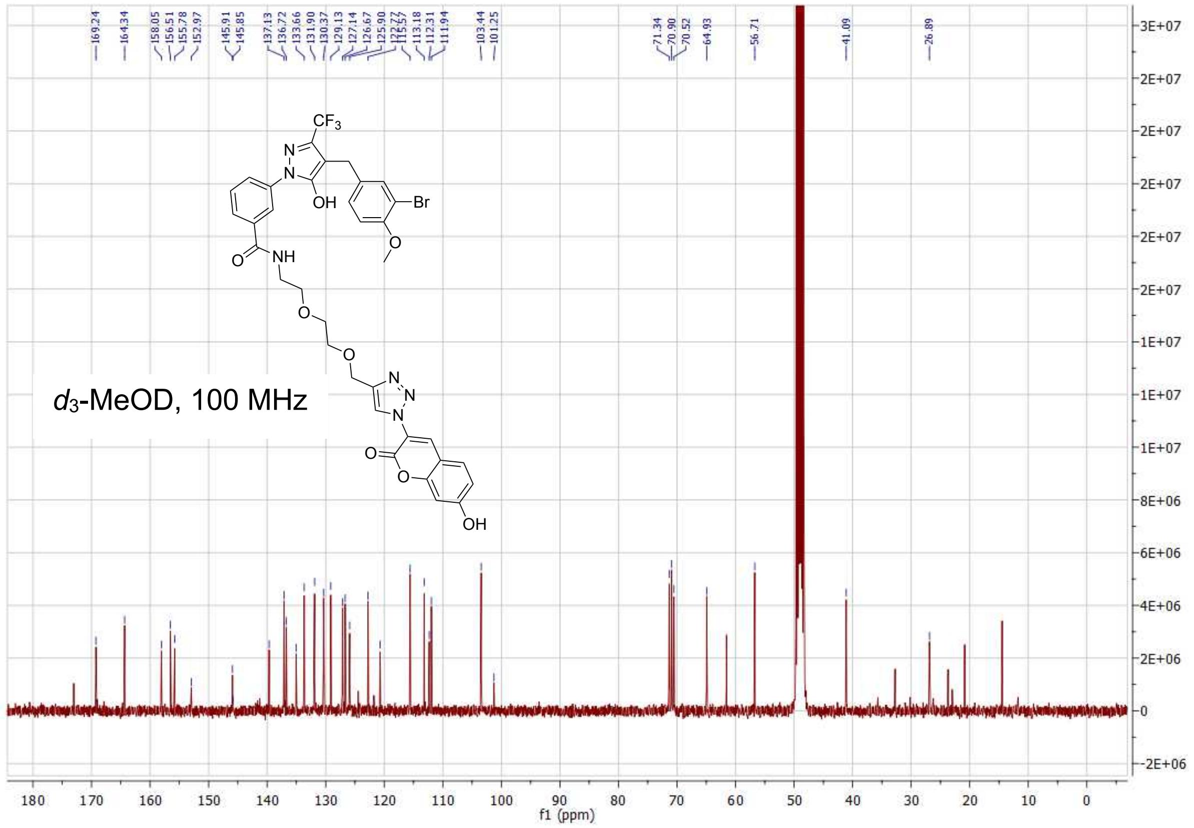

6a

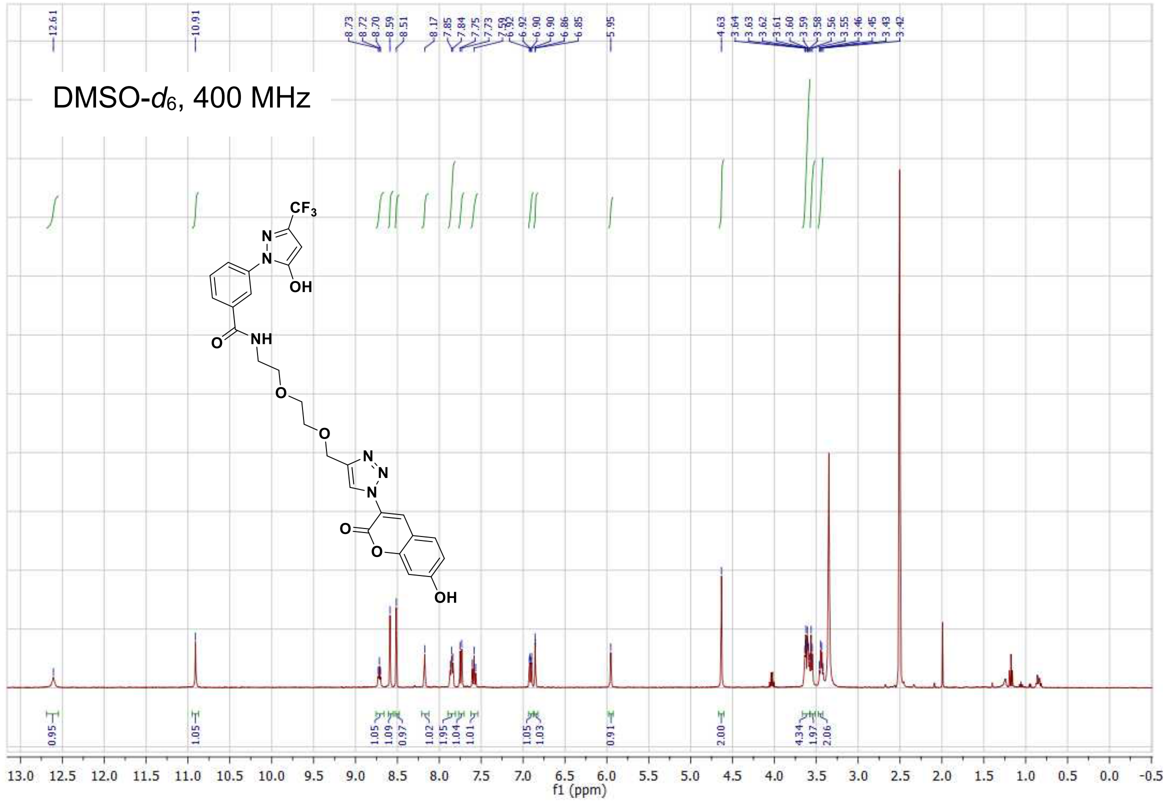

6b

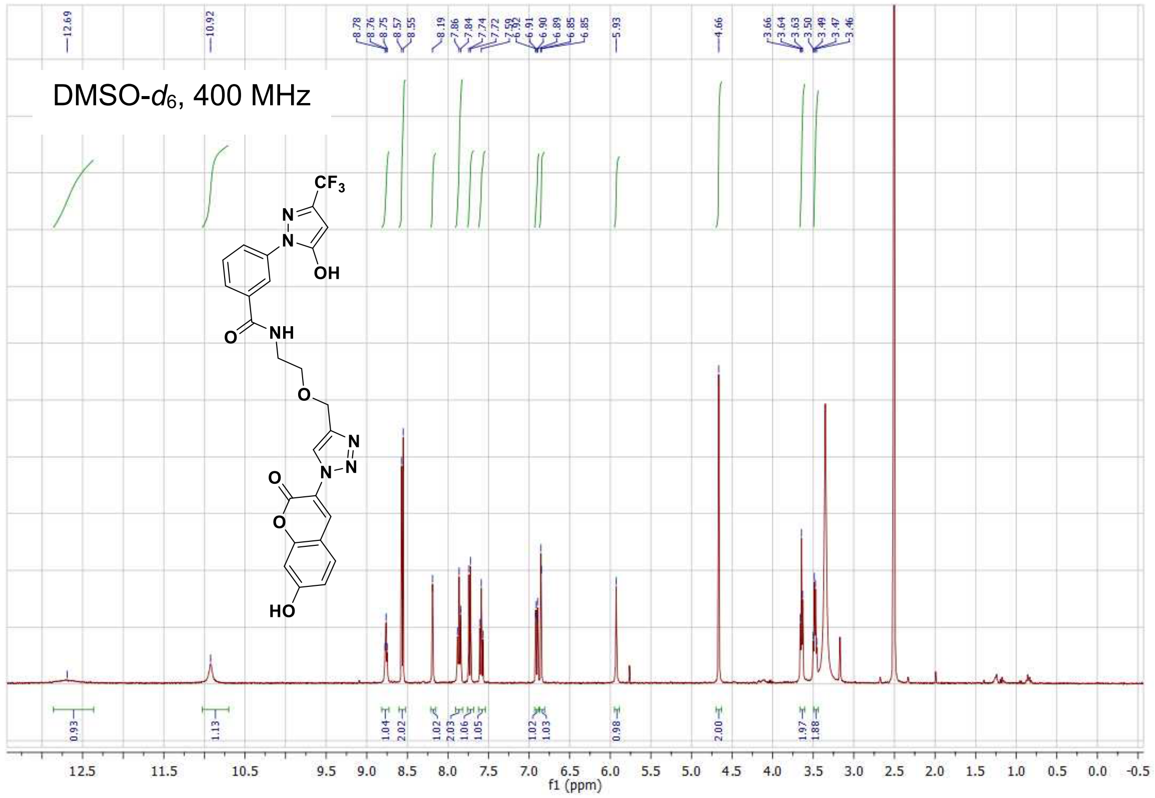

6c

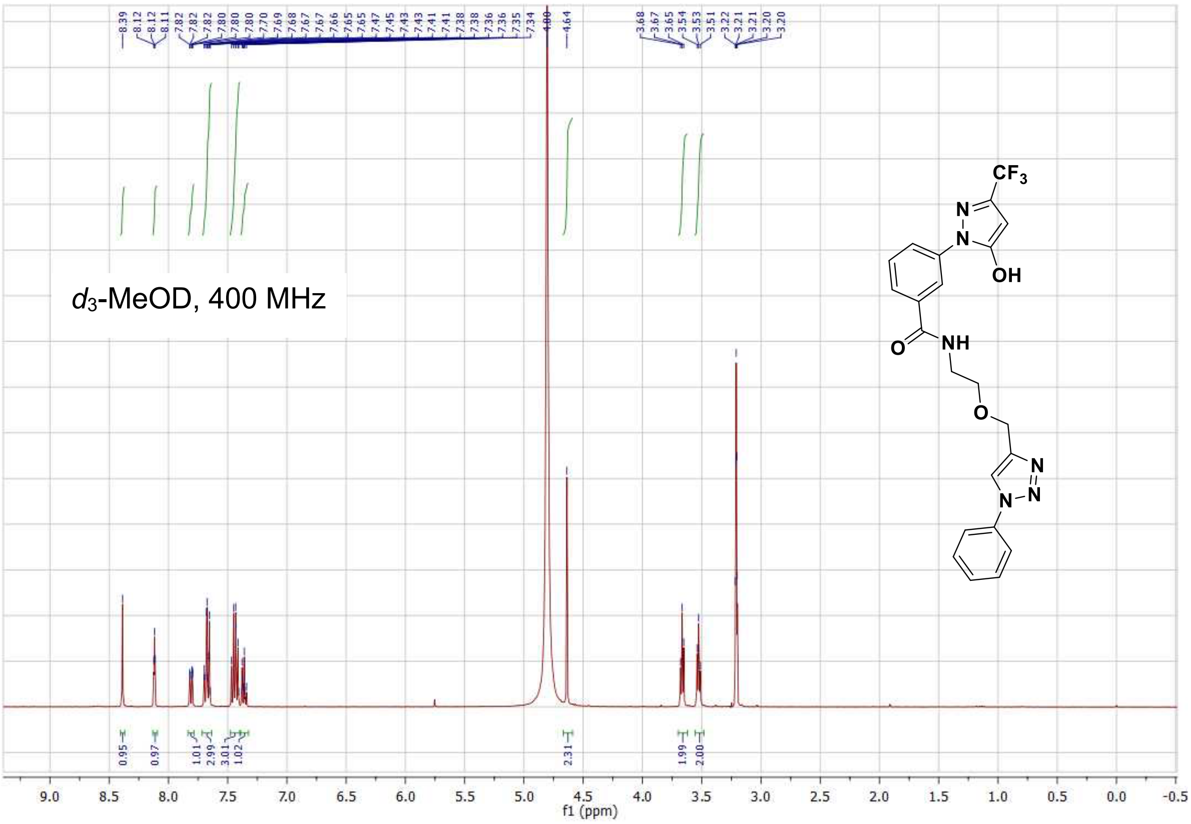

d3-MeOD, 400 MHz
CF3
N
N
OH
O
NH
O
N
N
N
f1 (ppm)

6d

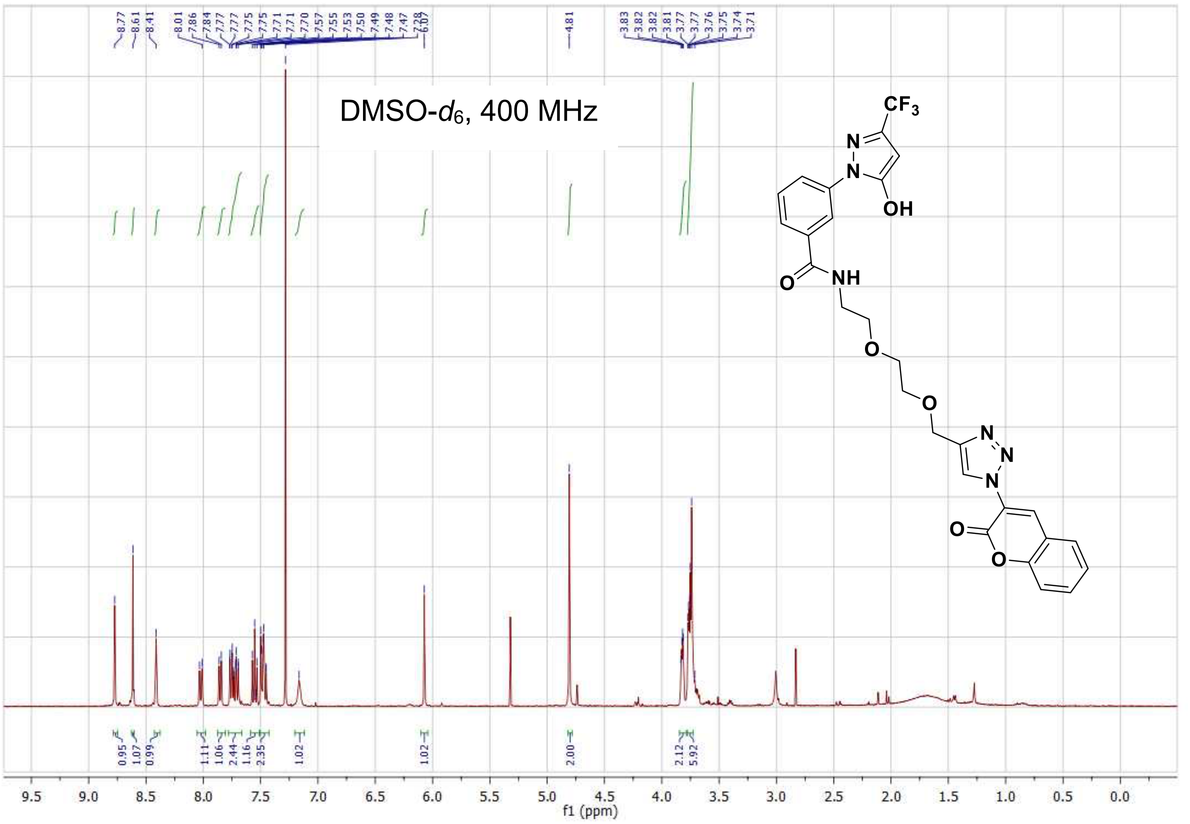

DMSO-d6, 400 MHz
CF3
N
N
OH
O
NH
O
O
N
N
N
O
O
f1 (ppm)

9a

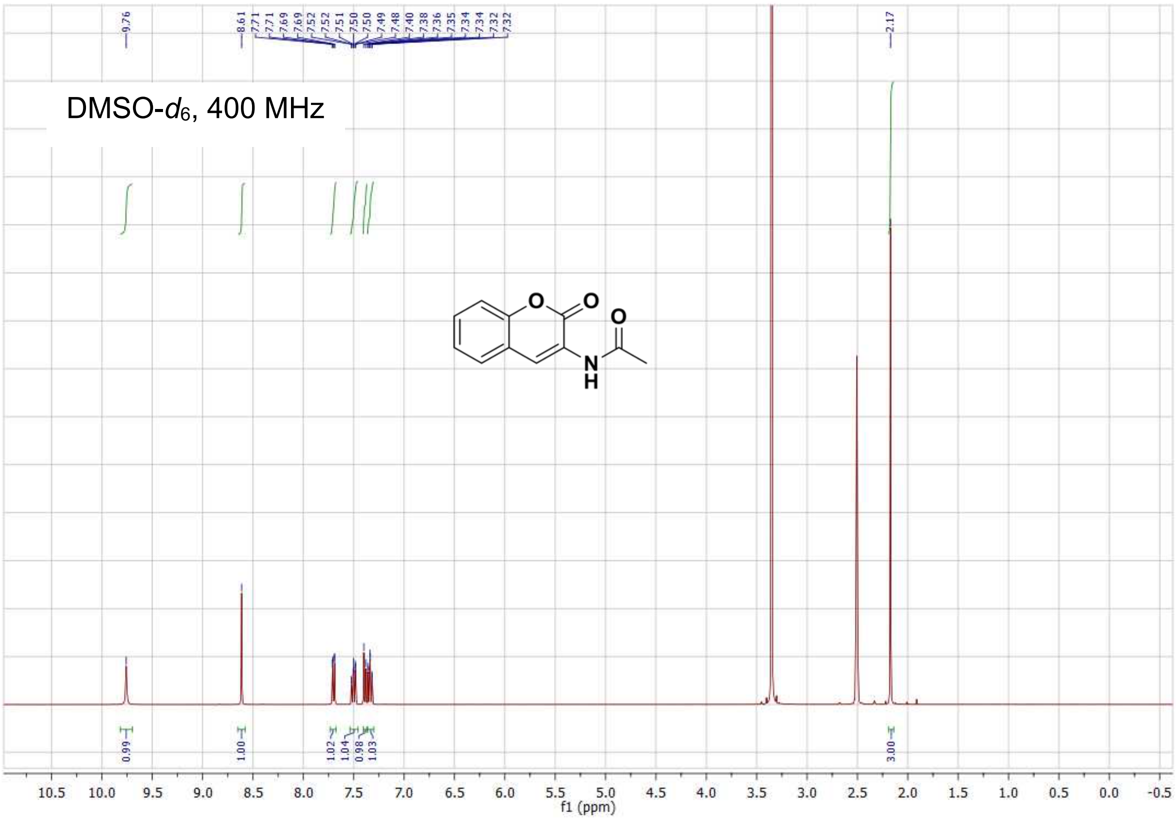

9.76
8.61
7.71
7.71
7.69
7.69
7.52
7.52
7.51
7.50
7.50
7.49
7.48
7.40
7.38
7.36
7.35
7.34
7.34
7.32
7.32
2.17
DMSO-d6, 400 MHz
O
O
O
N
H
0.99
1.00
1.02
1.04
0.98
1.03
3.00
10.5
10.0
9.5
9.0
8.5
8.0
7.5
7.0
6.5
6.0
5.5
5.0
4.5
4.0
3.5
3.0
2.5
2.0
1.5
1.0
0.5
0.0
-0.5
f1 (ppm)

9b

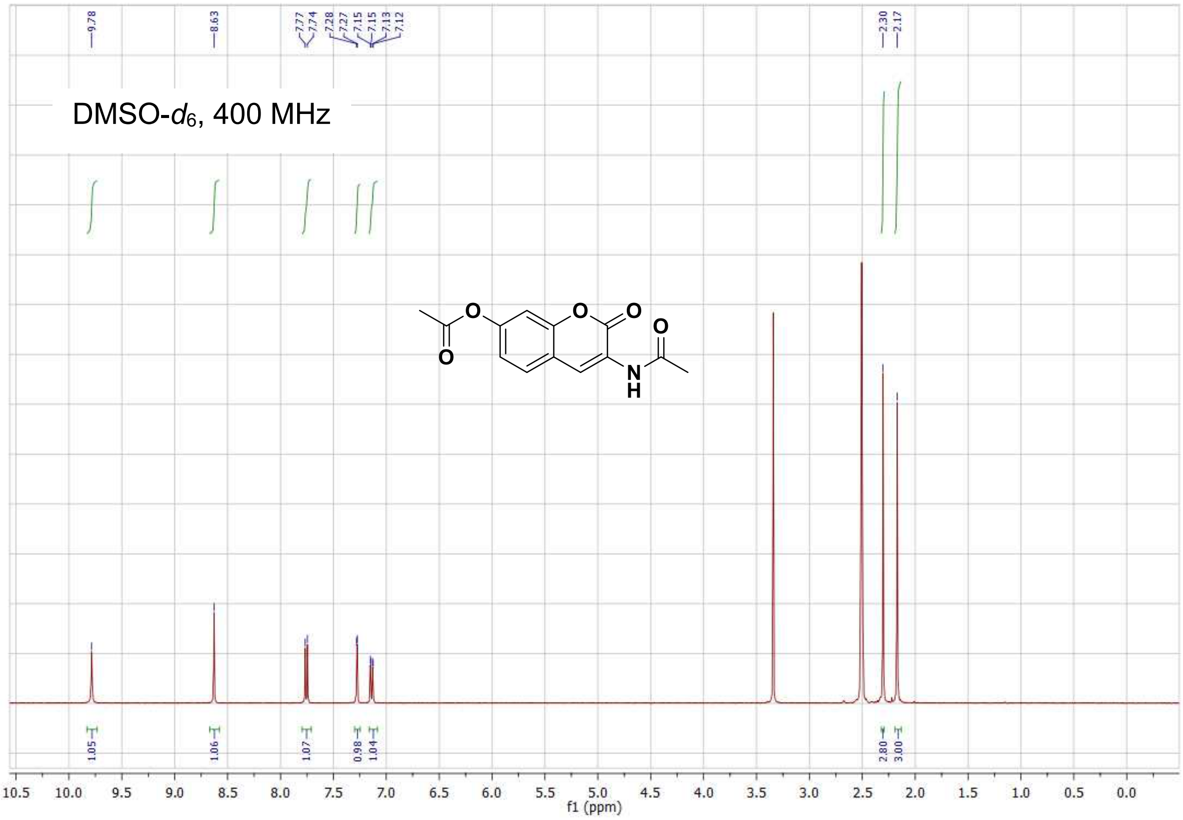

9.78
8.63
7.77
7.74
7.28
7.27
7.15
7.15
7.13
7.12
2.30
2.17
DMSO-d6, 400 MHz
O
O
O
O
O
N
H
1.05
1.06
1.07
0.98
1.04
2.80
3.00
10.5
10.0
9.5
9.0
8.5
8.0
7.5
7.0
6.5
6.0
5.5
5.0
4.5
4.0
3.5
3.0
2.5
2.0
1.5
1.0
0.5
0.0
f1 (ppm)

10a

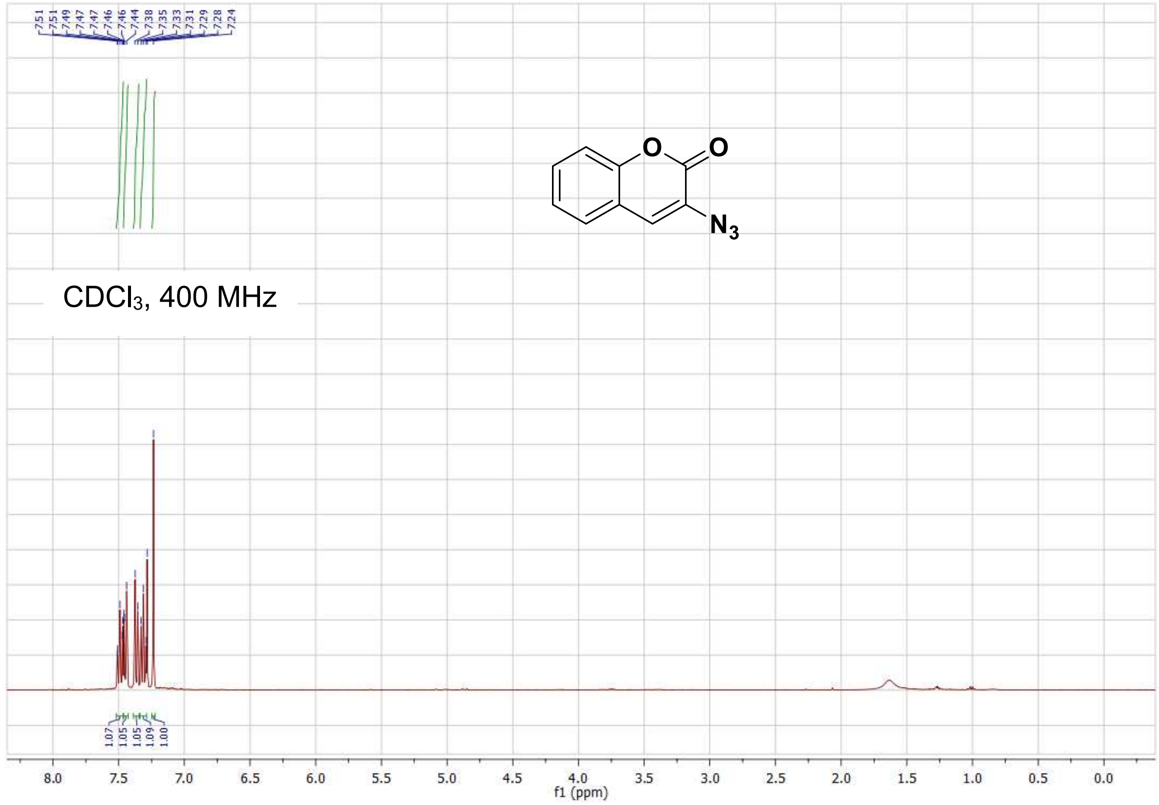
7.51
7.51
7.49
7.47
7.47
7.46
7.46
7.44
7.38
7.35
7.33
7.31
7.29
7.28
7.24
CDCl3, 400 MHz
O
O
N3
1.07
1.05
1.05
1.09
1.00
f1 (ppm)

10b

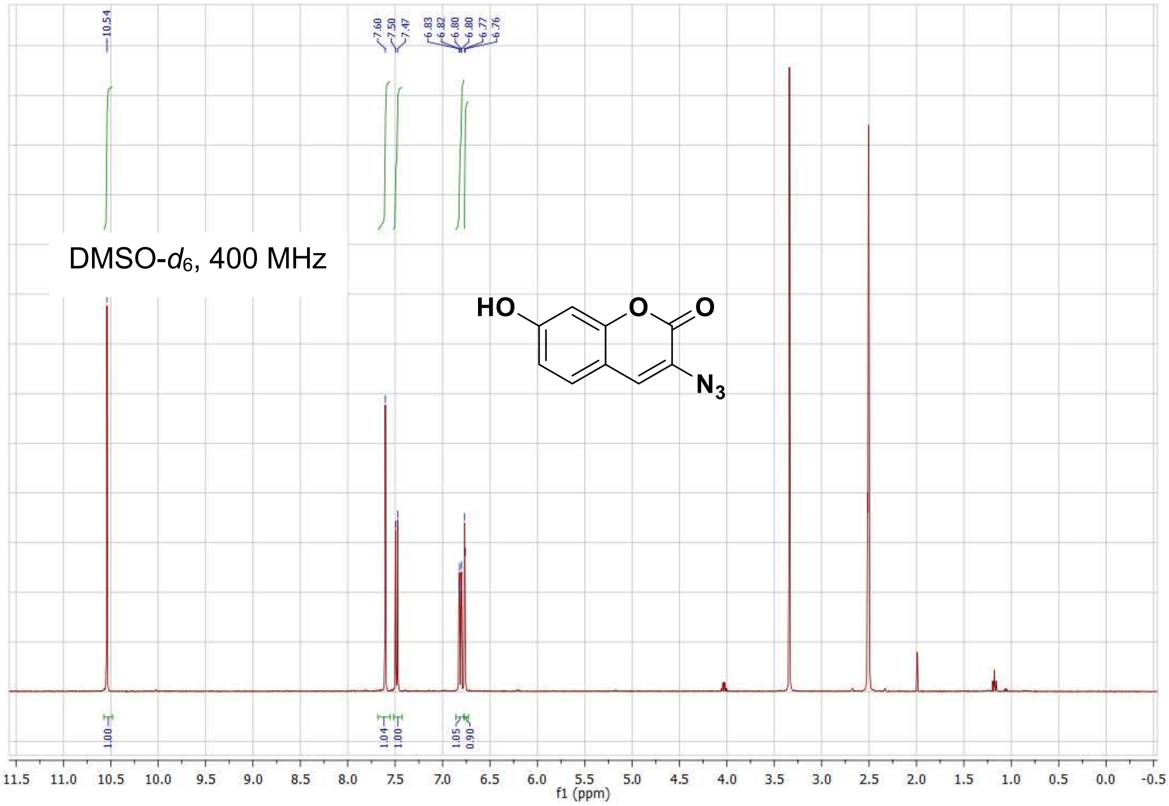
10.54
7.60
7.50
7.47
6.83
6.82
6.80
6.80
6.77
6.76
DMSO-d6, 400 MHz
HO
O
O
N3
1.00
1.04
1.00
1.05
0.90
f1 (ppm)
